# Modeling HIV-1 infection in the brain

**DOI:** 10.1371/journal.pcbi.1008305

**Published:** 2020-11-19

**Authors:** Colin T. Barker, Naveen K. Vaidya

**Affiliations:** 1 Department of Mathematics and Computer Science, Drury University, Missouri, USA; 2 Department of Mathematics and Statistics, University of Missouri-Kansas City, Missouri, USA; 3 Department of Mathematics and Statistics, San Diego State University, San Diego, California, USA; 4 Computational Science Research Center, San Diego State University, San Diego, California, USA; 5 Viral Information Institute, San Diego State University, San Diego, California, USA; ETH Zurich, SWITZERLAND

## Abstract

While highly active antiretroviral therapy (HAART) is successful in controlling the replication of Human Immunodeficiency Virus (HIV-1) in many patients, currently there is no cure for HIV-1, presumably due to the presence of reservoirs of the virus. One of the least studied viral reservoirs is the brain, which the virus enters by crossing the blood-brain barrier (BBB) via macrophages, which are considered as conduits between the blood and the brain. The presence of HIV-1 in the brain often leads to HIV associated neurocognitive disorders (HAND), such as encephalitis and early-onset dementia. In this study we develop a novel mathematical model that describes HIV-1 infection in the brain and in the plasma coupled via the BBB. The model predictions are consistent with data from macaques infected with a mixture of simian immunodeficiency virus (SIV) and simian-human immunodeficiency virus (SHIV). Using our model, we estimate the rate of virus transport across the BBB as well as viral replication inside the brain, and we compute the basic reproduction number. We also carry out thorough sensitivity analysis to define the robustness of the model predictions on virus dynamics inside the brain. Our model provides useful insight into virus replication within the brain and suggests that the brain can be an important reservoir causing long-term viral persistence.

## Author summary

Around the world HIV/AIDS remains among the most disastrous disease epidemics that humanity is facing. Currently several treatment regimens and drugs exist that can prolong the life of HIV infected individuals, but none can cure the disease. As the virus remains within infected individuals for life, including in their brain, a long lifespan leads to HIV associated neurocognitive disorders (HAND) such as encephalitis or early-onset dementia. Here we develop a mathematical model to study the viral dynamics in the brain. Our model, which can accurately describe experimental data from macaques, predicts a significantly high rate of HIV transport in and out of the brain through the blood-brain barrier (BBB). The possibility of ongoing HIV replication inside the brain predicted by our model indicates that the brain can be an important viral reservoir causing an obstacle to an HIV cure. This suggests that eradication of the virus inside the brain should also be considered while designing strategies for HIV cure.

## Introduction

Human Immunodeficiency Virus (HIV-1) constitutes a devastating epidemic the world faces today with nearly 37 million people currently living with the virus, and over one million annual deaths due to AIDS related illnesses [1]. Current therapy, namely highly active antiretroviral therapy (HAART), can successfully control viral loads in the plasma, and infected individuals may live nearly as long as uninfected individuals. However, no cure has yet been found despite continuous research and medical breakthroughs, presumably due to virus and/or viral proteins hiding in various reservoirs, such as the gut, the lungs, the liver, and the brain. Moreover, despite an undetected viral load in the plasma during HAART, many patients experience HIV associated neurocognitive disorders (HAND), such as encephalitis and early-onset dementia [2–5], mostly due to the extended period that infected individuals carry the virus supplied from the reservoirs. Among the viral reservoirs the brain represents the least studied one [3, 6–11], partly because of its association with the blood-brain barrier (BBB) and the difficulty of *in vivo* study on the brain infection. Thus it is important to gather insights into the viral dynamics in the brain to devise proper HIV-1 control strategies.

Recent studies have considered the virus in the brain as a major obstacle in the search for a cure [9, 12]. The brain has been recognized as a viral reservoir, but it still remains unclear whether or not viral replication occurs within the brain [4, 5, 10, 14]. Some effort has been made to suppress the virus within the brain, but the BBB drastically reduces the effectiveness of such treatment because many drugs cannot cross the BBB [3, 15]. Due to the difficulty in controlling HIV-1 in the brain as well as potential viral replication inside it, the brain can be an important reservoir causing an obstacle for a cure [6, 7, 16, 17]. There is a complex interplay between the viral dynamics of HIV-1 within the brain and within the plasma, and mathematical modeling may be able to uncover new insight.

Mathematical modeling has aided the study of within-host viral dynamics [18–20]. Unlike in the plasma, where HIV-1 primarily infects CD4+ T cells, the primary target cells for HIV-1 in the brain are macrophages [2, 21]. Immature macrophages (called monocytes) become infected by HIV-1 and penetrate the BBB before growing into mature infected macrophages [22, 23]. This method is often referred to as the Trojan-horse mechanism. CD4+ T cells less frequently cross the BBB, thus it is necessary to consider macrophages when studying HIV-1 in the brain, along with CD4+ T cells in the plasma. Currently existing viral dynamics models cannot explain these issues properly, and thus a new model coupling the plasma and the brain is needed to accurately explain the viral replication inside the brain.

In this study we develop a novel mathematical model to describe the HIV-1 viral dynamics within the brain. We identify key parameters by fitting our model to plasma and cerebral-spinal fluid (CSF) viral load data from an experiment using rhesus macaques infected with a mixture of Simian Immunodeficiency Virus (SIV) and Simian-Human Immunodeficiency Virus (SHIV). We consider three variants of the model to analyze whether viral replication within the brain occurs. We also explore the long-term stability of HIV-1 predicted by our model and determine its sensitivity to key parameters. Our study finds that the BBB plays a major role in the transport of HIV-1 from the brain to the plasma and vice versa, and that viral replication in the brain may partly explain the virus persistence despite ongoing HAART.

## Materials and methods

### Data

The data used in this study was obtained by digitizing results from published literature [24, 25]. In the published experiment [24, 25], three male rhesus macaques (*Macacamulatta*) were infected intravenously with a mixture of simian-human immunodeficiency virus (*SHIV*_*KU*−1*B*_ and *SHIV*_89−6*P*_) and simian immunodeficiency virus (*SIV*_17*E*−*Fr*_). These animals were monitored for a period of 12 weeks, and levels of circulating CD4+ *T* cells and viral loads in both the CSF and plasma were measured as described in Kumar *et al.* [25].

### Mathematical model

In the circulation (representing outside the brain), one of the primary target cells of HIV-1 are uninfected CD4+ T cells (*T*) [26]. These cells become infected (T*) by free virions (*V*) within the circulation at a rate *β*. Infected CD4+ T cells die at a rate *δ* per day and produce virions at a rate of *p* per day per infected cell. Uninfected *T* cells die at a rate *d* per day and are generated at a rate λ cells per day.

The major cells that HIV-1 infects in the brain are macrophages [8, 21]. To model this we include an uninfected population of macrophages (*M*) in the circulation that becomes infected (M*) upon interaction with free virus at a rate *β*_*M*_. These infected macrophages produce free virions at a rate *p*_*M*_ per day per infected cell and die at a rate of *δ*_*M*_ per day. Uninfected macrophages die at a rate of *d*_*M*_ per day and are generated at a rate λ_*M*_ cells per day. Note that the population of macrophages has been considered to contribute to viral persistence because of its longer lifespan [2, 8–10, 12, 13, 22, 27].

In order for a virion to enter the CSF in the brain it must pass through the BBB. It is not fully understood what factors modulate transit of HIV-1 RNA through the BBB into the CSF [12]. However studies show that the virus permeates the integrity of the BBB only via an infected macrophage [15, 21]. Clear mechanisms of how macrophages transport across the BBB are poorly understood. Since we are not modeling the BBB compartment separately, rather the BBB is considered as a barrier between two locations, inside and outside the brain, we model the transport (mobility) of macrophages across the BBB using a simple linear approach implemented widely in many studies, including lymphocytes movement in and out of blood [28] and virus infected lymphocyte movement in and out of follicular tissues in SIV-infected macaques [29]. We represent the rate of the macrophage transit through the BBB into the brain by *φ*. Macrophages are not known to generate independently within the brain [23]. The uninfected brain-macrophages become infected ($M_{B}^{*}$) by the virus in the brain [6, 11, 14, 22, 23] at a constant rate *β*_*M*_. These infected brain-macrophages produce free virions within the brain at a constant rate *p*_*M*_ per infected cell per day.

In this study, we consider the type of the virus inside the brain to be the same as the virus outside. However, the environment in the brain has been shown to alter the characteristics of free virions [3, 11, 23], and the viral load data from inside the brain and the outside the brain were measured separately. Therefore, we denote HIV-1 virions within the brain by a different variable *V*_*B*_. We assume that the free virions *V* and *V*_*B*_ are both cleared at the same per capita rate *c* per day. While limited evidence suggests the possible presence of HIV-1-infected T cells within the CSF [30], because the primary targets of HIV-1 within the brain are macrophages [2, 21], we consider only macrophages within the brain. Macrophages come out of the brain through the BBB into the bloodstream [31] at a constant rate *ψ*.

**Fig 1:**
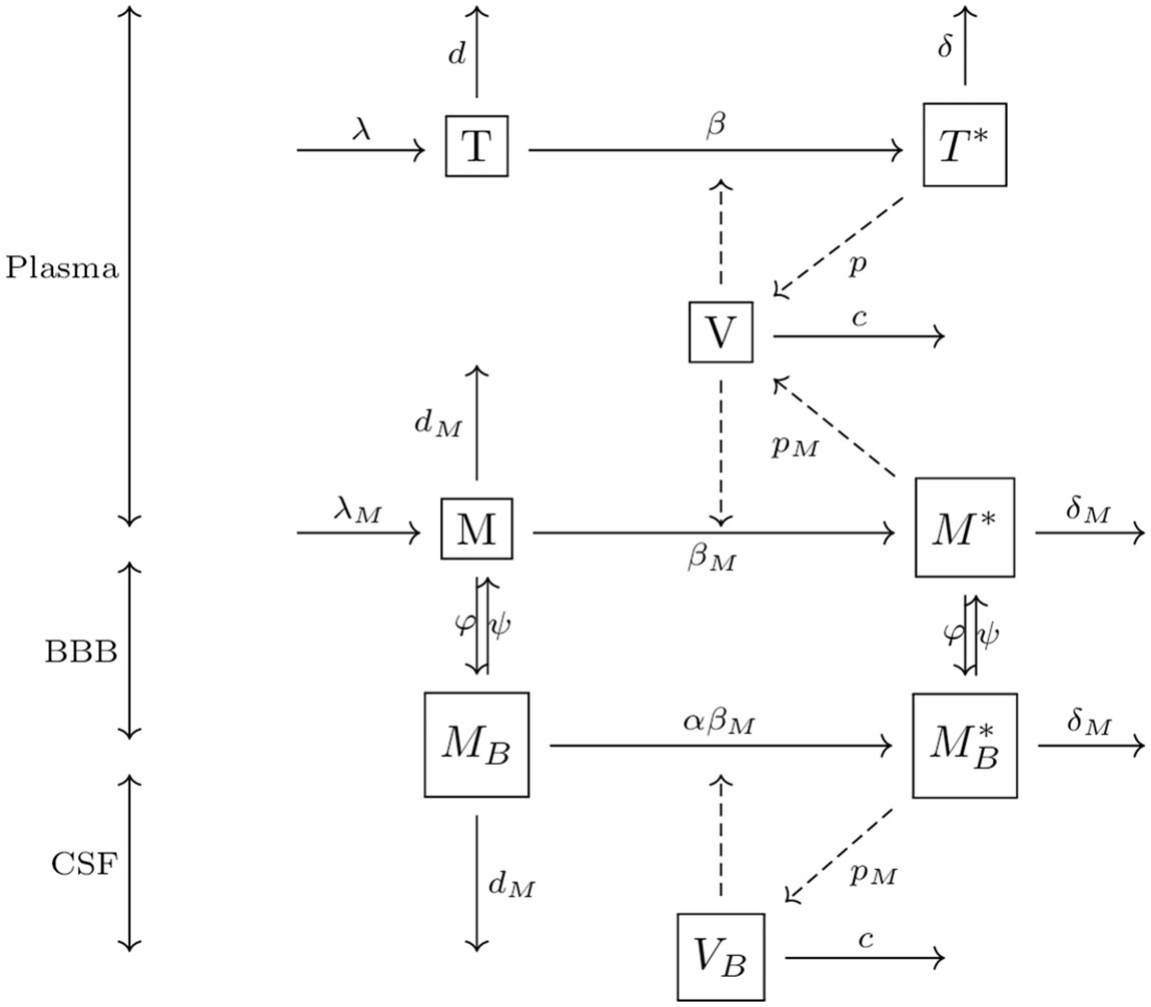
The schematic diagram of the model representing HIV-1 infection in the brain. The boxes represent a cell population, the solid arrows represent transport from one population to another, and the dashed arrows represent the cause for the corresponding events.

Considerable debate exists regarding whether or not viral replication occurs within the brain [6, 9, 10, 12, 14]. To perform deeper analysis from the modeling point of view, we develop three different variants of the model by introducing a parameter *α*, which represents the ratio between the infectivity of macrophages in the brain and outside of the brain. Model 1 (*α* = 1) assumes that viral replication occurs within the brain at the same rate as in the bloodstream. Similarly, Model 2 (*α* = 0) assumes that no viral replication occurs in the brain, and Model 3 (0 < *α* ≠ 1) assumes that the viral replication occurs at a different rate than outside of the brain. The schematic diagram of the model is shown in Fig 1. The model equations we use are as follows.

$$\begin{array}{c} \begin{array}{cc} \frac{\text{d}T}{\text{d}t} & =\lambda -\beta VT-dT,\\ \frac{\text{d}T*}{\text{d}t} & =\beta VT-\delta T^{*},\\ \frac{\text{d}M}{\text{d}t} & =\lambda_{M}+\psi M_{B}-\beta_{M}VM-\varphi M-d_{M}M,\\ \frac{\text{d}M*}{\text{d}t} & =\beta_{M}VM+\psi M_{B}^{*}-\varphi M^{*}-\delta_{M}M^{*},\\ \frac{\text{d}M_{B}}{\text{d}t} & =\varphi M-\psi M_{B}-\alpha \beta_{M}V_{B}M_{B}-d_{M}M_{B},\\ \frac{\text{d}M_{B}^{*}}{\text{d}t} & =\alpha \beta_{M}V_{B}M_{B}-\psi M_{B}^{*}+\varphi M^{*}-\delta_{M}M_{B}^{*},\\ \frac{\text{d}V}{\text{d}t} & =pT^{*}+p_{M}M^{*}-cV,\\ \frac{\text{d}V_{B}}{\text{d}t} & =p_{M}M_{B}^{*}-cV_{B}. \end{array} \end{array}$$ (1)

Three variants of the model are

Model 1: *α* = 1,
Model 2: *α* = 0,
Model 3: 0 < *α* ≠ 1.

### Parameter estimation and data fitting

We take *T*_0_ = 38700 as in Vaidya *et al.* [32]. From Haney *et al.* [33] we estimate *M*_0_ = 1463000 and *M*_*B*0_ = 20000. As estimated by Stafford *et al.* [20], the average life span of uninfected target T cells is 100 days, which implies *d* = 0.01 per day. Macrophages begin their life cycle as monocytes, and there are varying results regarding the age of the monocyte/macrophage lifespan ranging from three months to three years [23]. We take the average lifespan to be approximately 18 months, i.e. *d*_*M*_ ∼ 0.002 per day. As every macaque was uninfected at the beginning of the study, we take all infected cells to be zero, i.e., $T_{0}^{*}=M_{0}^{*}=M_{B0}^{*}=0$ [25]. As done in Vaidya *et al.* [32], we fix *p* based on the work of Chen *et al.* [34], who estimated the SIV burst size *in vivo* in rhesus macaques to be approximately 5 × 10^4^ virions per infected cell, and take *p* = 50, 000. Assuming a steady state before infection, we use λ = *dT*_0_ and λ_*M*_ = *d*_*M*_(*M*_0_ + *M*_*B*0_) to estimate λ and λ_*M*_. Schwartz *et al.* [35] estimated the rate of lentiviral production by an infected macrophage to be approximately 1000 virions per infected cell per day. Therefore, we set *p*_*M*_ = 1000 for our base case computation. The virion clearance rate during chronic infection in humans varies from 9.1 to 36.0 [36]. Thus we take the average *c* = 23 per day as the minimal estimate. However, we acknowledge that this rate may be higher in macaques.

We estimate the remaining parameters *β*, *β*_*M*_, *δ*, *δ*_*M*_, *φ*, *ψ* by fitting the model to the viral load data in the CSF and the plasma. We solve the system of ordinary differential equations (ODEs) numerically using the “ode15s” solver in MATLAB. The predicted log_10_ values were fitted to corresponding log-transformed viral load data using the nonlinear least squares regression, in which the sum of the squared residuals, that is, the difference between the model predictions and the corresponding experimental data, is minimized. We used the following formula to calculate the sum of the squared residuals:

$$\begin{array}{c} J=\frac{1}{N_{P}}\sum_{i=1}^{N_{P}}{\left[\log_{10}V\left(t_{i}\right)-\log_{10}\bar{V}\left(t_{i}\right)\right]}^{2}+\frac{1}{N_{B}}\sum_{i=1}^{N_{B}}{\left[\log_{10}V_{B}\left(t_{i}\right)-\log_{10}{\bar{V}}_{B}\left(t_{i}\right)\right]}^{2}, \end{array}$$ (2)

where *N*_*P*_ and *N*_*B*_ represent the total number of data points in the plasma and in the brain, respectively. *V* and $\bar{V}$, represent the virus concentrations in the plasma predicted by the model and those measured in the experimental data, respectively, while *V*_*B*_ and ${\bar{V}}_{B}$ represent the virus concentrations in CSF predicted by the model and those measured in the experimental data, respectively. For each best fit parameter estimate, we provide 95% confidence intervals (CI), which were computed from 500 replicates by bootstrapping the residuals [37, 38].

## Results

### Model selection

We fit the model to the data containing plasma viral load and the CSF viral load for each of the three monkeys. To compare models we used the Akaike information criterion (AIC) described by the following formula [39, 40].

$$\begin{array}{c} AIC=n\log(\frac{J}{n})+\frac{2n(N_{par}+1)}{n-N_{par}-2}, \end{array}$$ (3)

where *n* = *N*_*P*_ + *N*_*B*_ represents the total number of data points considered, *J* is the sum of the squared residuals (SSR), and *N*_*par*_ represents the number of parameters estimated through data-fitting. The SSR and the AIC values for each of Model 1, Model 2, and Model 3 are given in Table 1. Note that the lower the AIC value, the better the model fit. There is no significant difference in the AIC or SSR value between Model 1 and Model 2, but Model 3 has the highest AIC values (Table 1). Moreover, when the parameter *α* in Model 3 was fixed at some value other than 0 or 1, we did not get better AIC or SSR values than those from Model 1 and Model 2. This indicates that the extra parameter introduced in Model 3 did not improve the data fitting. The similar AIC values between Model 1 and Model 2 suggest that the available data is not enough to decide whether viral replication occurs or does not occur within the brain. However, Model 1 is supported by the previous study by Schnell [13], who identified the virus replication inside the brain of HIV-infected patients and indicated that HIV replication in the central nervous system (CNS) contributes to neurocognitive decline. Therefore, we select Model 1 to present the subsequent results in the sections to follow. For comparison purposes, the results using Model 2 are also presented in S2 Text. In general, the patterns and overall behavior of Model 2 (S2 Text) are similar to Model 1, with some differences in predicted quantitative values.

**Table 1:** SSR and AIC values for each of Model 1 (*α* = 1), Model 2 (*α* = 0), and Model 3 (0 < *α* ≠ 1) fitted to each of the three monkeys.

	Model 1 (*α* = 1)		Model 2 (*α* = 0)		Model 3 (0 < *α* ≠ 1)	
	SSR	AIC	SSR	AIC	SSR	AIC
**Monkey 1**	4.6235	17.1562	4.4903	16.7469	3.8943	26.8866
**Monkey 2**	2.4144	55.788	2.4163	55.7966	2.4159	145.7951
**Monkey 3**	4.9797	18.1952	4.9758	18.1841	4.9781	30.3239

**Fig 2:**
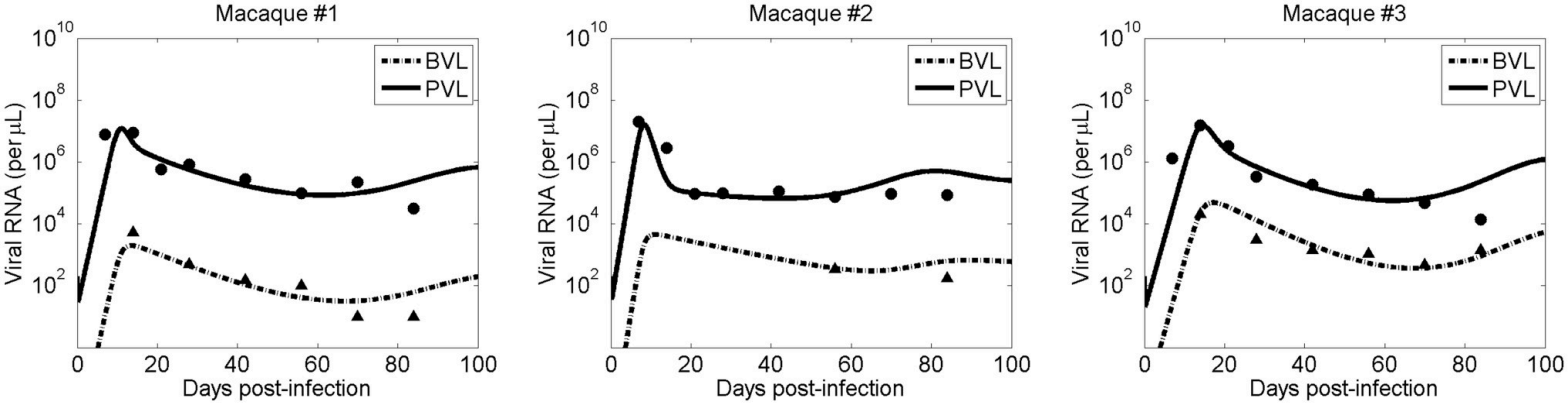
Model fit to the data. Plasma viral load (solid line) and CSF viral load (dashed line) predicted by the selected model, i.e. Model 1, along with the experimental data (filled circle: plasma viral load; filled triangle: CSF viral load) from three monkeys [24, 25].

The predictions of the selected model, i.e. Model 1, along with the data for each of the three monkeys are shown in Fig 2. Our model agrees well with the data (Fig 2). The data fitting of Model 2 and Model 3 is also provided in S1 Fig. The estimated parameters are given in Table 2.

**Table 2:** Parameter estimates through data fitting. Estimated parameters from fitting the selected model, i.e. Model 1, to each of the three monkey’s data. Paired values in parentheses represent 95% confidence intervals.

	*δ*	*δ*_*M*_	*φ*	*ψ*	*β*	*β*_*M*_
	day^−1^	day^−1^	day^−1^	day^−1^	ml/day	ml/day
Monkey 1	1.7319 (0.5555,1.8049)	0.2067 (0.1405,0.4141)	0.0117 (0.00220,0.22342)	9.4052 (8.2458,10.8779)	3.7332E-8 (1.9456E-8,7.4280E-8)	1.0018E-9 (9.9297E-10,1.0000E-9)
Monkey 2	1.6129 (0.8940,1.8214)	0.0673 (0.0234,0.1256)	0.78565 (0.33675,2.3305)	15.0023 (14.4669,15.2483)	4.4009E-8 (3.5322E-8,7.3811E-8)	4.0068E-11 (1.0000E-11,4.0119-11)
Monkey 3	1.0766 (0.5941,1.1664)	0.2127 (0.1550,0.2797)	0.29149 (0.08801,0.91395)	8.8010 (8.6176,9.0556)	2.5809E-8 (1.8701E-8,2.6271E-8)	6.9003E-10 (3.5739E-10,9.3840E-10)

### Rates of infection and cell death

We estimated that the rate, *β*, at which the virus infects CD4+ T cells, ranges between 2.58 × 10^−8^ and 4.40 × 10^−8^ viral RNA copies per *μ*L per day. These estimates are consistent with the previous estimates [26]. The infection rate estimated for macrophages, *β*_*M*_, ranges between 4.01 × 10^−11^ and 1.00 × 10^−9^ viral RNA copies per *μ*L per day, implying that macrophages are less susceptible to viral infection than CD4+ T cells. Similarly, we found that the death rate of infected macrophage (median *δ*_*M*_ ∼ 0.21 per day) is significantly lower than the death rate of infected CD4+ T cells (median *δ* ∼ 1.61 per day). Thus our model suggests that infected macrophages persist with the virus far longer than infected T cells, which is consistent with findings from previous experiments [22, 23, 41].

### Reproduction number

The basic reproduction number ($R_{0}$) is defined as the average number of secondary infected cells produced by a single infected cell when there is no target cell limitation [42]. In viral dynamics, the basic reproduction number is an important threshold that can determine whether infection occurs. Specifically, if $R_{0}<1$ the infection dies out, and if $R_{0}>1$ the infection occurs [42]. For our model we use the next-generation method [42, 43] to compute $R_{0}$. The details for this computation as well as an explicit formula for $R_{0}$ are given in S1 Text.

**Fig 3:**
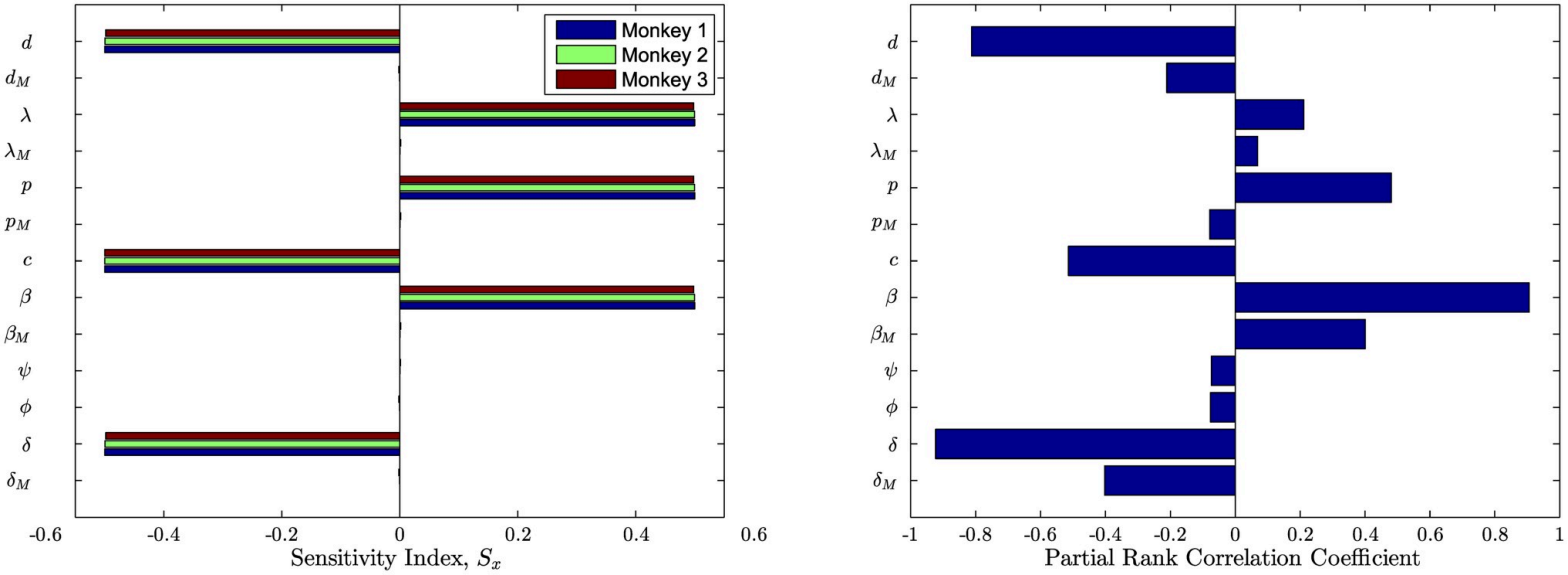
Sensitivity of parameter estimations to $R_{0}$. Local sensitivity of $R_{0}$ based on sensitivity index (left) and the partial rank correlation coefficients for global sensitivity of $R_{0}$ based on Latin Hypercube sampling (right).

Using the parameters estimated above in the $R_{0}$ formula, we obtained the basic reproduction number for each monkey. We found that $R_{0}$ ranges from 1.33 to 1.55. Note that $R_{0}>1$ in each case as expected because the experimental data show that the infection persists in each monkey. We further perform local sensitivity analysis to identify how sensitive the value of $R_{0}$ is to each parameter. To quantify the sensitivity we considered the sensitivity index *S*_*x*_ [44], given by

$$S_{x}=(\frac{x}{{ℜ}_{0}})(\frac{\partial {ℜ}_{0}}{\partial x}),$$

where *x* is a parameter whose sensitivity is sought. Based on the *S*_*x*_ values (Fig 3), we identified that the parameters *d*, *β*, *p*, *c*, *δ*, and λ have the greatest influence (*S*_*x*_ ∼ 0.5) on $R_{0}$, whereas *φ*, *ψ*, *d*_*M*_, *β*_*M*_, *p*_*M*_, *δ*_*M*_, λ_*M*_ have much less effect (*S*_*x*_ ∼ 1 × 10^−4^). We observe that the parameters greatly influencing $R_{0}$ are mostly *T* cell related. Thus the *T* cell and related parameters are primary contributors to the initial establishment of the viral infection. We now extend the analysis to the global sensitivity by computing the partial rank correlation coefficients for Latin Hypercube sampling from the global parameter space. In the global parameter space, we observe that although macrophage-related parameters can also significantly affect the basic reproduction number, the most strongly correlated parameters remain those associated with T cells.

### Transport through the BBB

Regarding infection in the brain, the transport of virus through the BBB plays a critical role. These mechanisms can be studied through the parameters *φ* and *ψ* of our model. Our estimates show that the per capita rate of macrophage entry into the brain, *φ* ∼ 0.29 per day, is significantly less than the per capita rate of macrophage exit from the brain, *ψ* ∼ 9.41 per day (Table 2). This implies that it is possible for the transport of virus out of the brain via infected macrophages to be greater than the transport of virus into the brain, but the net flow of virus also depends on the amount of infected macrophages outside and inside the brain. As a result, the amount of virus, which replicates inside the brain and then exits into the bloodstream through the BBB, can be significantly high. Thus the brain may act as an HIV-1 reservoir causing the persistent infection despite control of virus in the bloodstream through successful treatment.

**Fig 4:**
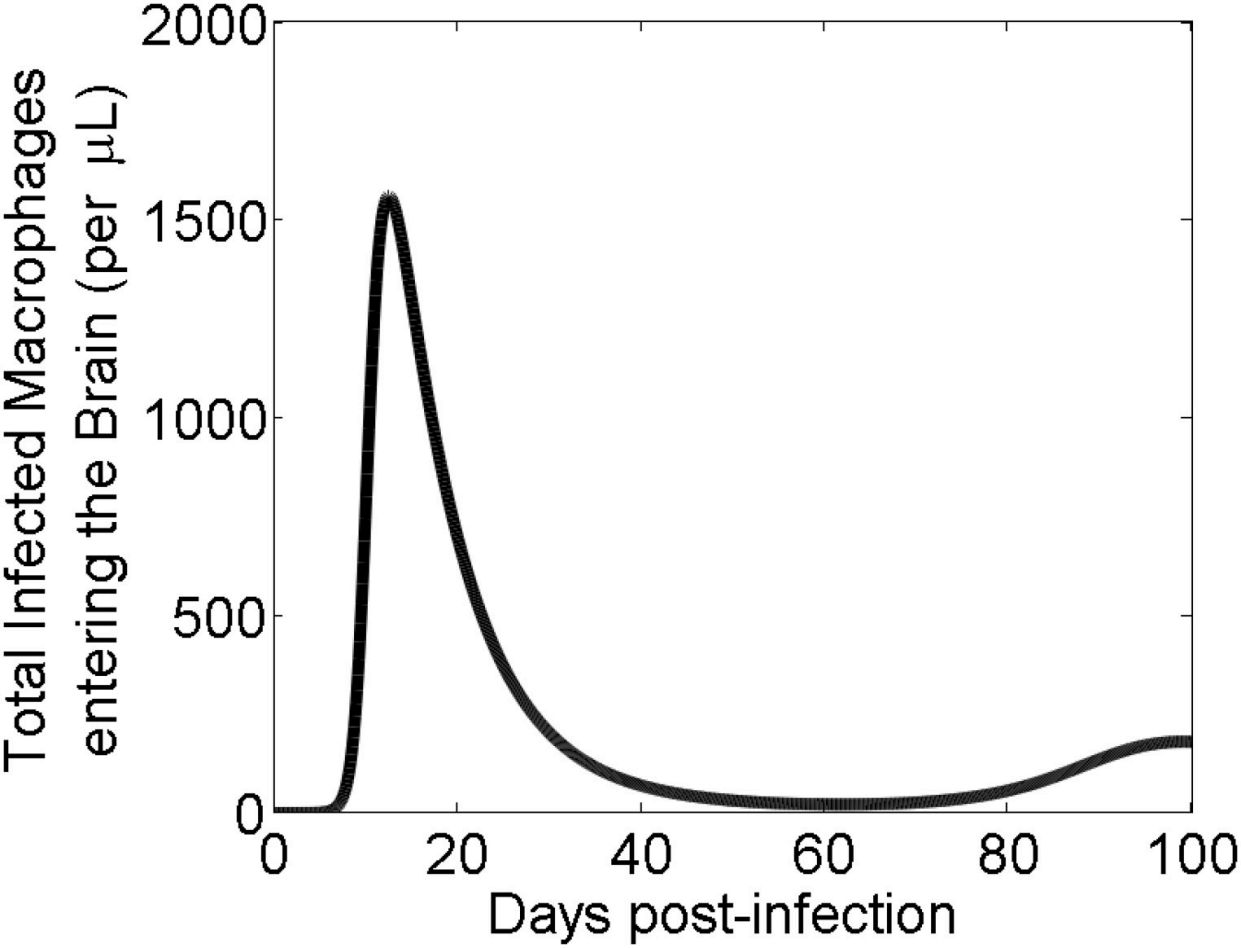
Incoming infected macrophages entering the brain (φM*). Model simulations of the total count of infected macrophages (φM*) entering the brain for 100 days post-infection.

Because of potential selection imposed by the BBB, especially for the entry of virus into the brain, we ask a question whether inflow of the virus into the brain is constant and thus whether the brain compartment can be studied in isolation as done in some previous studies [17]. To analyze viral entry into the brain we calculated the rate of the number of infected macrophages (φM*) entering into the brain over time for 100 days post-infection (Fig 4). The model prediction suggests that infected macrophages enter the brain through the BBB at time-varying rate, depending upon the infection outside the brain. This indicates that in order to accurately predict the viral dynamics in the brain, both the brain and the plasma must be considered as one coupled system rather than two separate ones, at least during the acute phase of infection.

### Cell and virus dynamics

**Fig 5:**
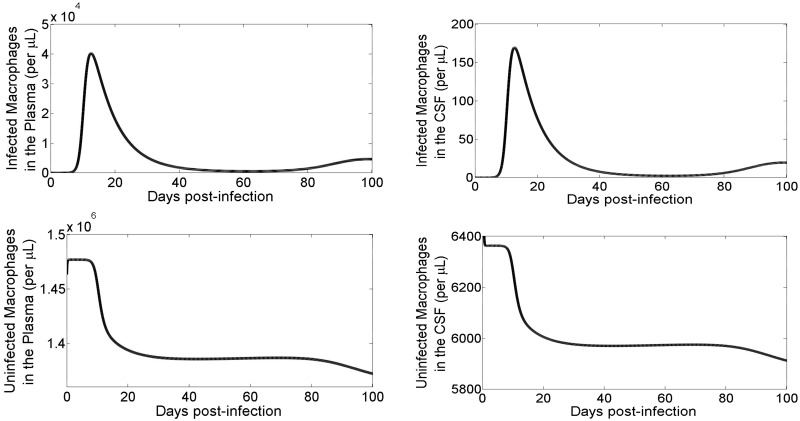
Simulations of macrophages in the plasma and the CSF. Model simulations over 100 days post-infection of infected macrophages (top row) and uninfected macrophages (bottom row) in the plasma (left column) and in the brain (right column).

We first used our model to study the acute phase dynamics of macrophages (Fig 5). The infected macrophages in the plasma and the brain both reach a peak at approximately 18 days post-infection, and then decline steadily over the next three weeks, eventually reaching a set point level. The dynamics of infected macrophages in the brain is similar to that of the infected macrophages in the plasma, however the amount of infected macrophages in the brain is significantly lower (peak at ∼170 per *μL*) than the infected macrophages in the plasma (peak at ∼40, 000 per *μL*). The uninfected macrophages, both in the brain and in the plasma, decline rapidly (by ∼6%) from their initial amounts.

**Fig 6:**
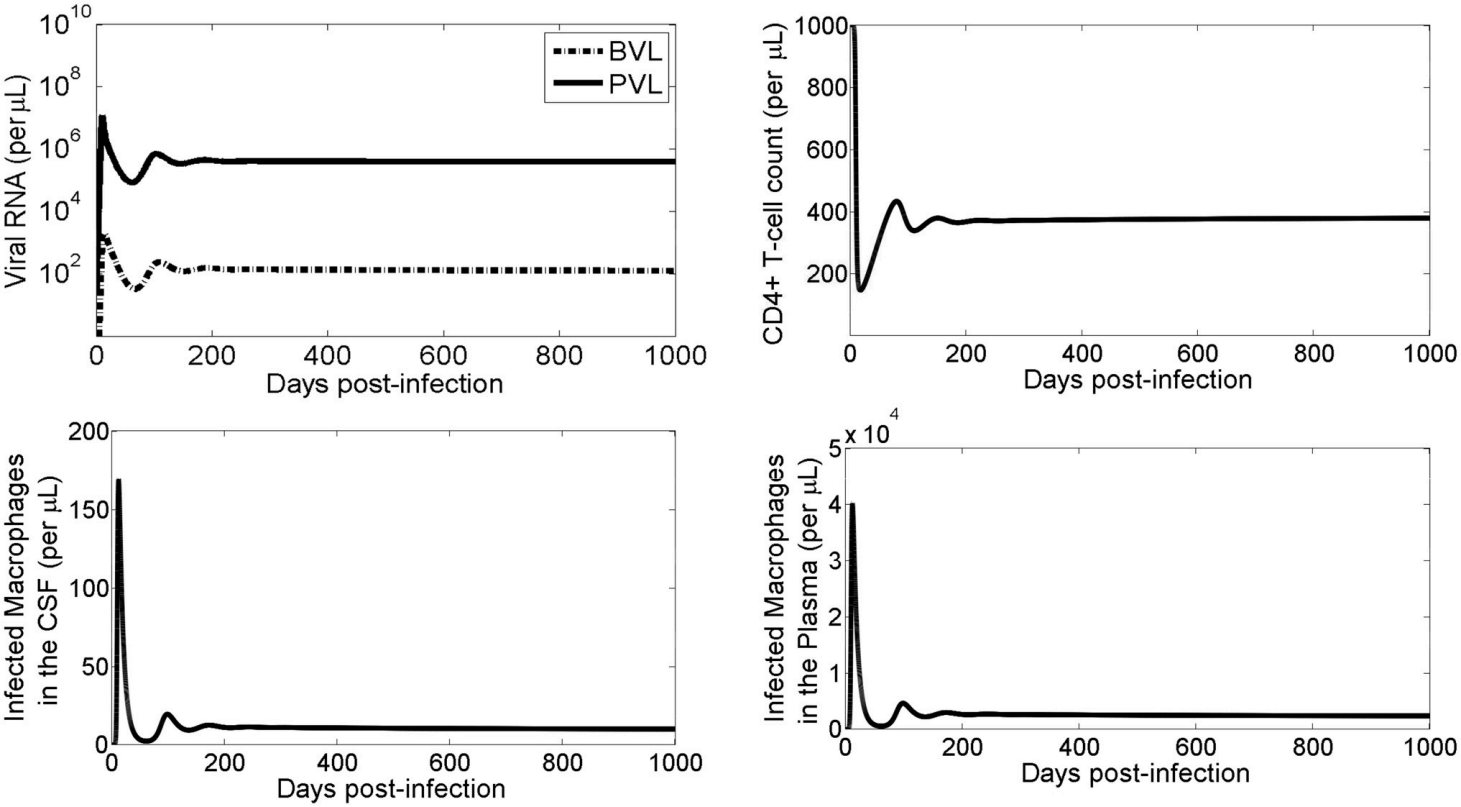
Long-term model simulations. Viral load (top left) for the plasma (solid line) and the brain (dashed line) along with the CD4+ T cell count (top right) and the total infected macrophages (bottom row) in the brain (bottom left) and in the plasma (bottom right).

We also studied the long-term dynamics by performing model simulations for 1000 days (approximately 3 years). After approximately 200 days the CD4+ T cell count, the infected macrophages in the brain and the plasma, and the viral RNA copies in the brain and the plasma all reach a steady state (Fig 6). The steady state level of the infected macrophages in the brain is roughly one fourth of that outside the brain (200 per *μL* outside vs 50 per *μL* inside the brain). Similarly, the steady-state level of viral RNA in the brain is nearly three-fold less than that in the plasma (∼10^3^ vRNA copies in the brain vs. ∼10^6^ vRNA copies in the plasma), consistent with the experimental results [25]. The CD4+ T cell count drops rapidly and levels off at 400 shortly after day 200.

As predicted by our model, the small amount of infected macrophages hiding inside the brain as well as ongoing replication of the virus inside the brain may partially contribute to the low level of viral persistence during the treatment of infected patients since many drugs cannot enter the brain through the BBB [3]. To further study the impact of the virus produced in the brain, we simulated viral dynamics under HAART, in which the viral production outside the brain was completely suppressed (*p* = *p*_*M*_ = 0 outside the brain) and the viral production inside the brain was allowed (*p*_*M*_ > 0 inside the brain). We found that for low levels of viral production both the brain and plasma viral load remain undetected, but for high level viral production the brain viral load becomes detected while the plasma viral load remains undetected (S3 Text). Therefore, while in the absence of treatment, the virus supplied from the brain may not significantly contribute to the plasma viral load because of high viral production outside the brain, in the presence of treatment, the contribution of the ongoing viral replication and production in the brain to the persistence of virus can be remarkably high.

### Sensitivity analysis

#### Sensitivity of data-fitting estimates on the fixed parameters

Our data-fitting estimates were based on the fixed values of parameters *M*_0_, *M*_*B*0_, *d*_*M*_, and *p*_*M*_. While we estimated values of these parameters from the literature, there is uncertainty with these values. Therefore, we performed sensitivity analysis of the data-fitting parameter estimates to the choice of the initial conditions *M*_0_ and *M*_*B*0_ and the choice of *d*_*M*_ and *p*_*M*_ (S2 and S3 Figs).

First, we performed 200 different data fittings using *M*_0_ and *M*_*B*0_ values chosen randomly from the uniform distribution between 10% less and 10% more values than the base value. We observed that the median change in the estimated parameters remained below 10% for each parameter and for each monkey except for *β*_*M*_ in Monkey 2 (22% change) (S2 Fig). This high sensitivity of *β*_*M*_ for Monkey 2 is likely due to the lack of enough data points in the brain for this monkey. The overall mean change of each estimated parameter also remained less than 10% from the base case estimate, suggesting our estimates were robust within these ranges of *M*_0_ and *M*_*B*0_.

Then, we performed 200 data fittings using *d*_*M*_ and *p*_*M*_ values sampled randomly from the values between 10% less and 10% more than the base values. In this case, we observed that both the median and the mean change in the estimated parameters never exceeded more than 8% for each parameter for each monkey. This suggests that our parameter estimates for *d*_*M*_ and *p*_*M*_ were also robust within these ranges for *d*_*M*_ and *p*_*M*_.

#### Sensitivity of model dynamics on the general parameter space

**Fig 7:**
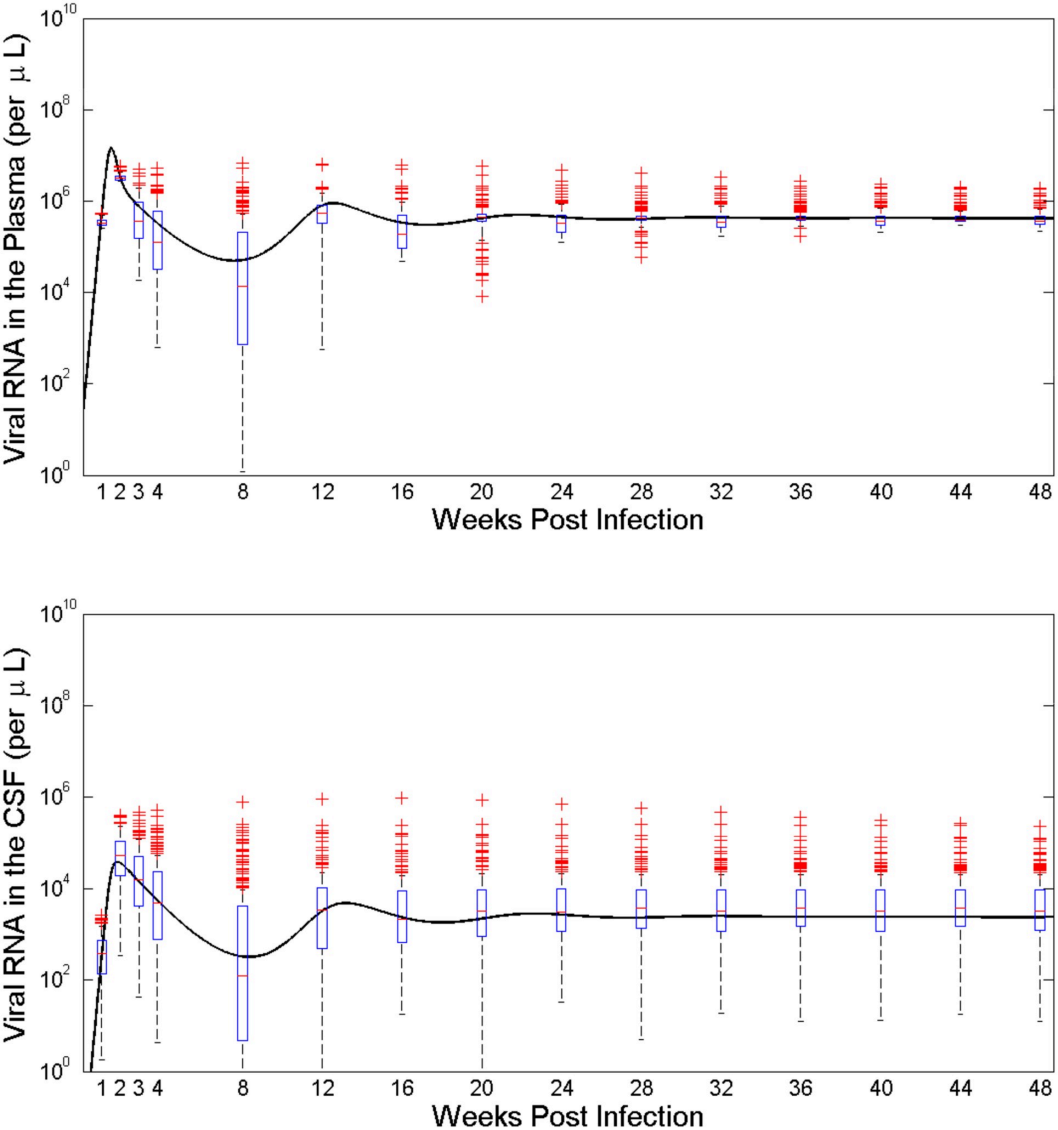
Box-plots of the results of 200 simulations of the model from Latin hypercube sampling. The sensitivity of the dynamics of plasma viral load (top) and the CSF viral load (bottom) based on 200 Latin Hypercube sampling. The black solid line represents the viral dynamics predicted by the model with median parameters estimated from three monkey data.

**Fig 8:**
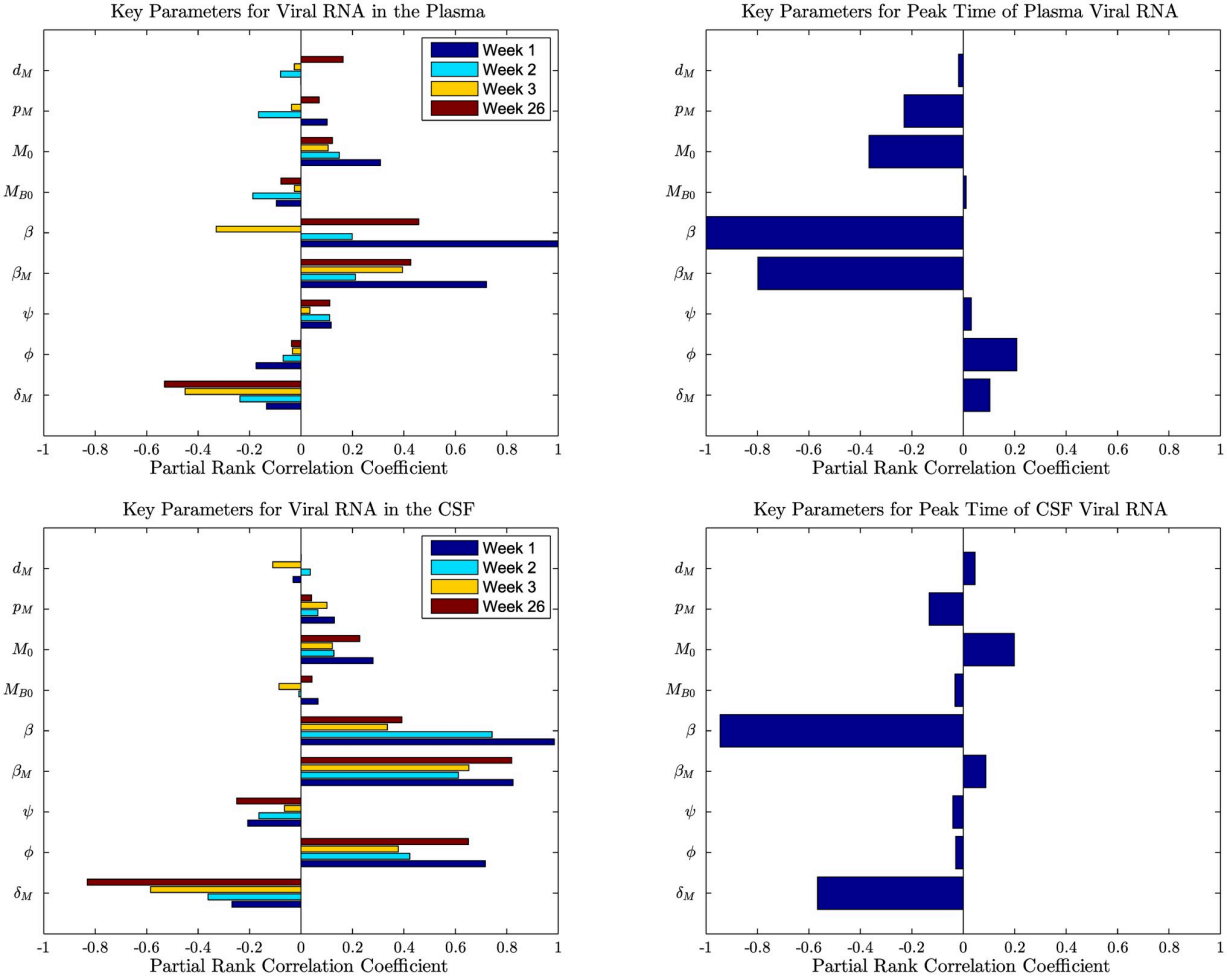
Partial rank correlation coefficients from the Latin hypercube sampling method. PRCC values of the plasma (top, left) and the CSF (bottom, left) viral loads at weeks 1 (pre-peak), 2 (peak), 3 (post-peak), and 26 (set-point) post infection along with the PRCC values of the timing of the peak viral loads in the plasma (top right) and in the CSF (bottom right).

Given the limited number of data sets and extreme complications for the study of brain virus, the results based on the model dynamics from our limited estimates require further analysis on a wider parameter space. To examine the robustness of our model dynamics we performed 200 simulations using a Latin hypercube sampling (LHS) of nine parameters (*δ*_*M*_, *ψ*, *φ*, *β*_*M*_, *β*, *M*_*B*0_, *M*_0_, *p*_*M*_, and *d*_*M*_). The box-plots and partial rank correlation coefficients (PRCC) of this sensitivity analysis is shown in Figs 7 and 8, respectively. The dynamics from the data fitting estimates (solid lines) are clearly captured within the boxes of the LHS results. Predicted dynamics are more sensitive to the parameters during early part of the infection. Variation of the viral dynamics in the brain is much wider than that in the plasma (Fig 7).

We calculated PRCC values at weeks one, two, three, and 26, corresponding to the timings for pre-peak, peak, post-peak, and set point viral load, respectively (Fig 8). The computed partial rank correlation coefficients indicate that parameters, in general, have stronger correlation to the viral load in the CSF compared to that in the plasma. Both plasma and CSF viral load are most correlated with parameters related to infection rates, *β*_*M*_ and *β*, and macrophage life-span, *δ*_*M*_. In addition, the CSF viral load is highly correlated with the BBB related parameter, *φ*. These parameters, except *δ*_*M*_, mainly have larger effect on early viral load than on late viral load. Both plasma and CSF viral loads are positively impacted by *β*_*M*_ and *β*, and negatively impacted by *δ*_*M*_, while *φ* has positive impact on CSF viral load and negative impact (but with smaller magnitude) on the plasma viral load.

We also computed PRCC values for the timing of the peak viral loads in the plasma and the CSF (Fig 8). Out of the nine parameters sampled, only the parameters *β*, *φ*, and *p*_*M*_ impact the timing of the peak viral load in the CSF and the plasma in the similar manner (i.e., either both positive correlation or both negative correlation), with *β* being the most impactful parameter. In the case of the other parameters, there is an opposite effect on the timing of peak viral loads in the plasma compared to the CSF.

## Discussion

HIV-1 remains a major public health challenge and one of the leading causes of death worldwide [1]. While HIV-1 is one of the most studied diseases, viral dynamics in the brain remains one of the least studied aspects of the disease. In particular, the transport of the virus through the BBB and the presence of ongoing viral replication in the brain are poorly understood. To gain insights into these issues, here we developed a mathematical model that can explain the experimental viral load data in the plasma and the CSF from SIV/SHIV infected macaques. Using our model and experimental data we estimated key parameters, including those related to the BBB. In addition, we performed thorough sensitivity analyses, including with Latin Hypercube sampling, to examine the robustness of the dynamics described by our model. Our model predicts a number of interesting results that may be helpful to control the virus in the brain, thereby potentially reducing the occurrence of HAND since HIV replication in the brain can contribute to neurocongnitive decline [14].

Our model predicts that the entry of HIV virus and/or viral protein via macrophages crossing the BBB is time-varying in nature and the rate of entry may depend on the virus dynamics outside the brain. This shows that while the chronic phase HIV dynamics in the brain may be studied with the brain compartment in isolation, as done in some previous studies [17], the modeling study for acute phase HIV-1 dynamics in the brain should include both the brain and the plasma as one coupled system. This underscores the importance of getting deeper insights into the BBB and viral transport across it.

In addition to the virus entering into the brain from outside, our model comparison on the basis of AIC values reveals that there is a possibility of ongoing viral replications and production of new virus inside the brain. However, the infection rate of macrophages, the major target cells for viral replication inside the brain, is significantly smaller than that of CD4+ T cells. This implies that macrophages are less susceptible to HIV-1 than CD4+ T cells, but once infected they remain so for a much longer time as indicated by our estimate of a significantly lower death rate of infected macrophages than of infected CD4+ T cells. As a result of these infections outside and inside the brain, our model predicts that in the long run the virus in the brain reaches a steady-state nearly three-fold lower than the virus in the plasma. Similarly, there can be persistence of infected macrophages in the brain with a steady state level significantly lower than the infected cells in the plasma. This indicates that without treatment the virus maintains infectiousness throughout an individual’s lifetime not only in the plasma, but also in the brain. This long-term persistence of the virus inside the brain is likely linked to HAND including early-onset dementia and encephalitis [2–4, 11, 12].

Importantly, our estimates show that the rate of viral exit from the brain, *ψ*, is significantly higher than the rate of viral entry into the brain, *φ*. It should, however, be noted that the time dependent net flow of the virus also depends on the available infected macrophages carrying viral RNAs. This flow rate of the virus across the BBB combined with persistent low level ongoing viral replication and viral production inside the brain indicate that the brain can be an important reservoir supplying virus to the bloodstream. Because of the high viral load outside the brain compared to the viral load in the brain (3-fold lower in the brain) in untreated individuals, the contribution of the virus coming out of the brain may not be significant to the plasma viral load for untreated individuals. However, this contribution can be extremely important for viral persistence in the presence of HAART (S3 Text). Since many antiretroviral drug molecules cannot enter the brain through the BBB [3], viral replication can continue in the brain despite suppression of the virus to undetected levels in the plasma, thereby causing an obstacle to the cure of HIV through treatment. Upon treatment interruption, the virus produced in the brain may contribute to further replication outside the brain resulting in the viral rebounds. Therefore, antiretroviral agents that can obstruct the replication inside the brain are necessary for successful control of HIV-1 infection.

We also computed the basic reproduction number, $R_{0}$, for each monkey, and found that the value of $R_{0}$ (1.33 to 1.55) is consistent with previous estimates [26]. Furthermore, we performed a sensitivity analysis to identify the parameters most affecting $R_{0}$. Our results show that those parameters related closely to T cells are the most impactful for determining the value of $R_{0}$, and thus best characterize the initial infection. This suggests that the brain has minimal effect on the initial infectiousness of HIV-1. This result is consistent with the facts that infection initiates outside the brain first, and it takes some time for the virus to penetrate the BBB and enter the brain [12].

We acknowledge several limitations of our study. Our parameter estimates are based on a limited number of macaques. Furthermore, the available data was not enough for our model to determine whether viral replication occurs in the brain. Also, note that the infection in these macaques was initiated using a mixture of different viruses (*SHIV* and *SIV*), thus the obtained results, including computed $R_{0}$ values, should be interpreted as the combined effect of these viruses in the mixture. More data with infection from individual virus types is necessary to identify whether the results remain the same for the single virus type. We considered only macrophages as targets of HIV-1 inside the brain. However, brain macrophages may differentiate into microglia. Also, a small amount of CD4+ T cells may exists within the brain [30] and other cells such as astrocytes may be HIV-1 targets. We did not consider the immune responses, which might be particularly important for the long-term dynamics. Since the primary objective of this study was to analyze viral dynamics inside the brain and the data did not include other potential viral reservoirs, we considered only the brain and the circulation in our model. While our study provided important insights into the potential role of the brain as a viral reservoir, other important reservoirs, such as Infected Resting Cells [13, 45, 46], should also be considered to accurately describe the viral persistence. For such study, we require the data collected simultaneously from other reservoirs as well. Moreover, a detailed study of macrophage transport across the BBB is needed to properly model the transport mechanism to accurately evaluate the impact of the BBB. Further experimental and theoretical study could also examine the persistent HIV-1 replication in the brain while individuals are under ongoing treatment.

In summary, we developed a novel mathematical model to examine the viral load dynamics in the plasma and the CSF of HIV-1. From this model and experimental data we estimated key parameters related to the BBB and viral transport through the BBB which may indicate the behavior of the brain as a virus reservoir. Results from this model may help offer insight into better ways to control HIV-1, and thus reducing the development of HAND.

## Supporting information

S1 Fig Data fitting of Model 2 and Model 3. Plasma viral load (solid line) and CSF viral load (dashed line) predicted by Model 2 (left column) and Model 3 (right column), along with the experimental data (filled circle: plasma viral load; filled triangle: CSF viral load) from three monkeys. While the graphs showed are comparable for the fitting of these models, the AIC values calculated for Model 2 are similar to Model 1, but the AIC values calculated for Model 3 are significantly high. Therefore the extra parameter introduced in Model 3 did not improve the data fit.
(PDF)

S2 Fig Sensitivity of *M*_0_ and *M*_*B*0_. Box-plots of the parameter estimates from 200 data-fittings with values of *M*_0_ and *M*_*B*0_ selected randomly from ±10% of the base values. Each subfigure represents the result for one of the parameters estimated. We found that the estimated values remain almost the same when *M*_0_, and *M*_*B*0_ were chosen from the range of ±10% of the base values.
(PDF)

S3 Fig Sensitivity of *d*_*M*_ and *p*_*M*_. Box-plots of the parameter estimates from 200 data-fittings with values of *d*_*M*_ and *p*_*M*_ selected randomly from ±10% of the base values. Each subfigure represents the result for one of the parameters estimated. We found that the estimated values remain almost the same when *d*_*M*_ and *p*_*M*_ were chosen from the range of ±10% of the base values.
(PDF)

S1 TextDerivation of $R_{0}$.(PDF)

S2 Text Results from Model 2. (PDF)

S3 Text Viral dynamics under HAART. (PDF)
