# Supplementary figures and images for: Modeling HIV-1 infection in the brain
Source: PLoS Comput Biol. 2020 Nov 19;16(11):e1008305. doi: 10.1371/journal.pcbi.1008305 (PMC7714358; doi:10.1371/journal.pcbi.1008305)

Macaque # 1, Model # 2

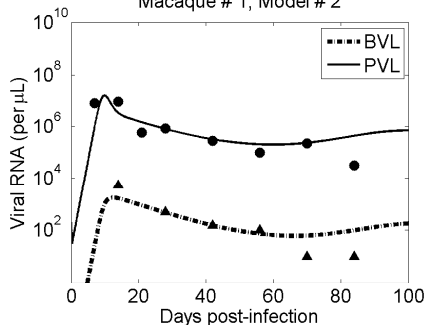

Macaque # 1, Model # 3

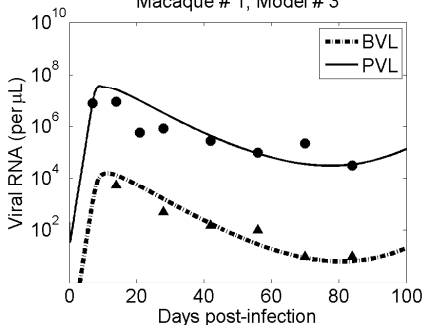

Macaque # 2, Model # 2

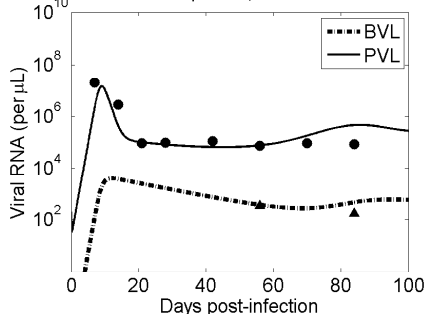

Macaque # 2, Model # 3

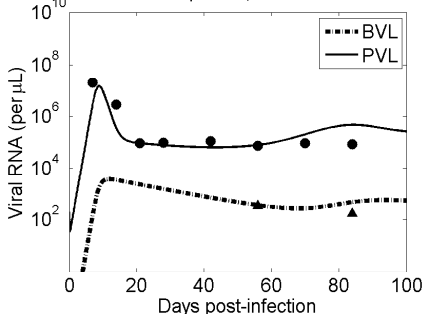

Macaque # 3, Model # 2

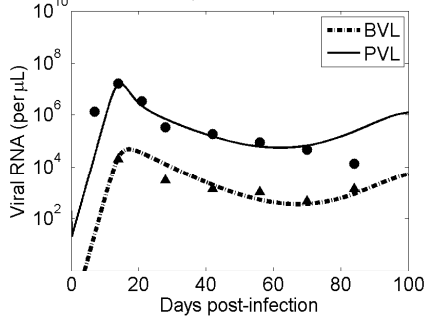

Macaque # 3, Model # 3

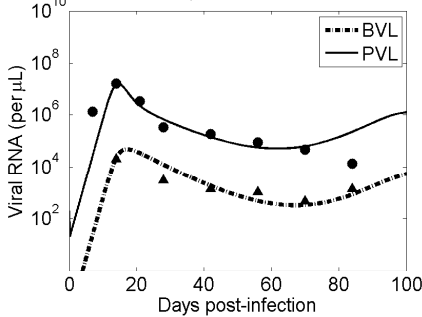

Supplement: S1 Fig — Plasma viral load (solid line) and CSF viral load (dashed line) predicted by Model 2 (left column) and Model 3 (right column), along with the experimental data (filled circle: plasma viral load; filled triangle: CSF viral load) from three monkeys. While the graphs showed are comparable for the fitting of these models, the AIC values calculated for Model 2 are similar to Model 1, but the AIC values calculated for Model 3 are significantly high. Therefore the extra parameter introduced in Model 3 did not improve the data fit. (PDF) [file pcbi.1008305.s001.pdf]

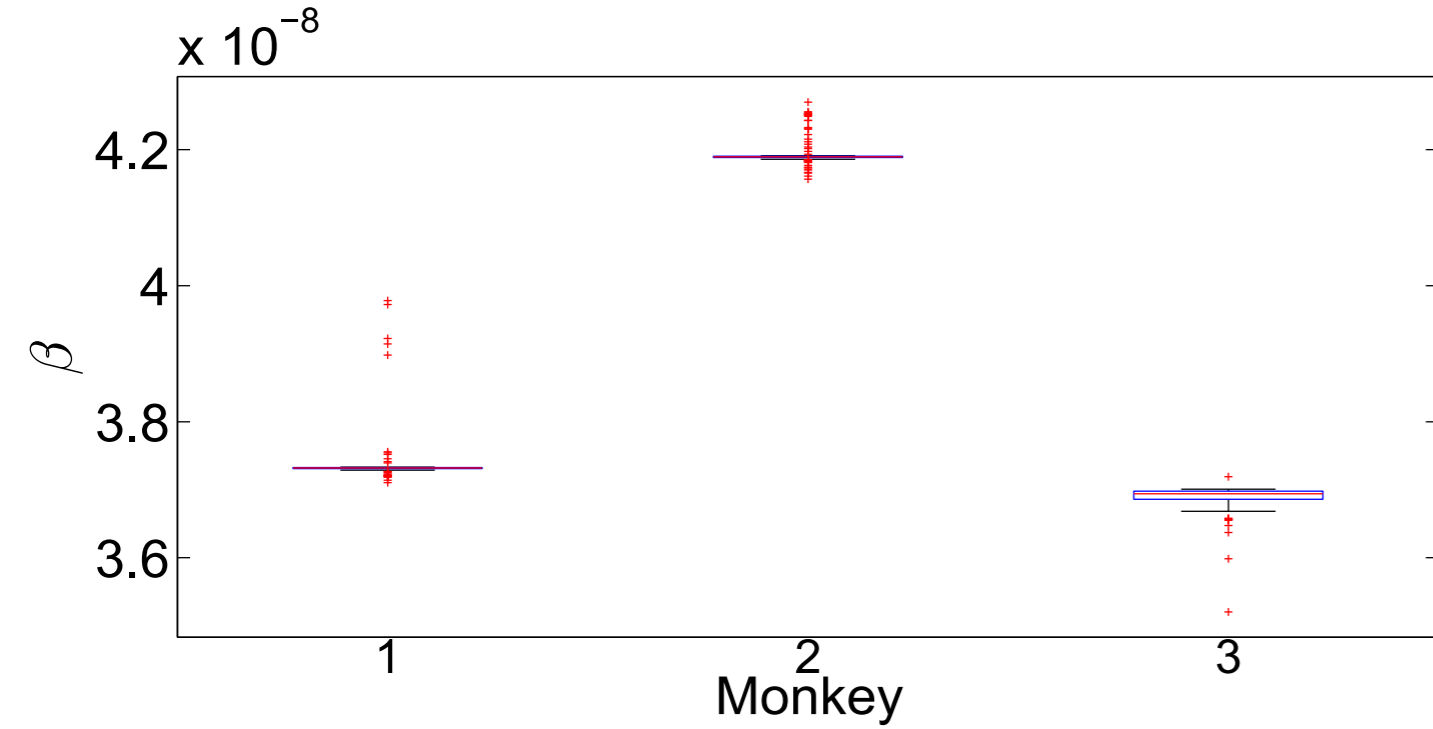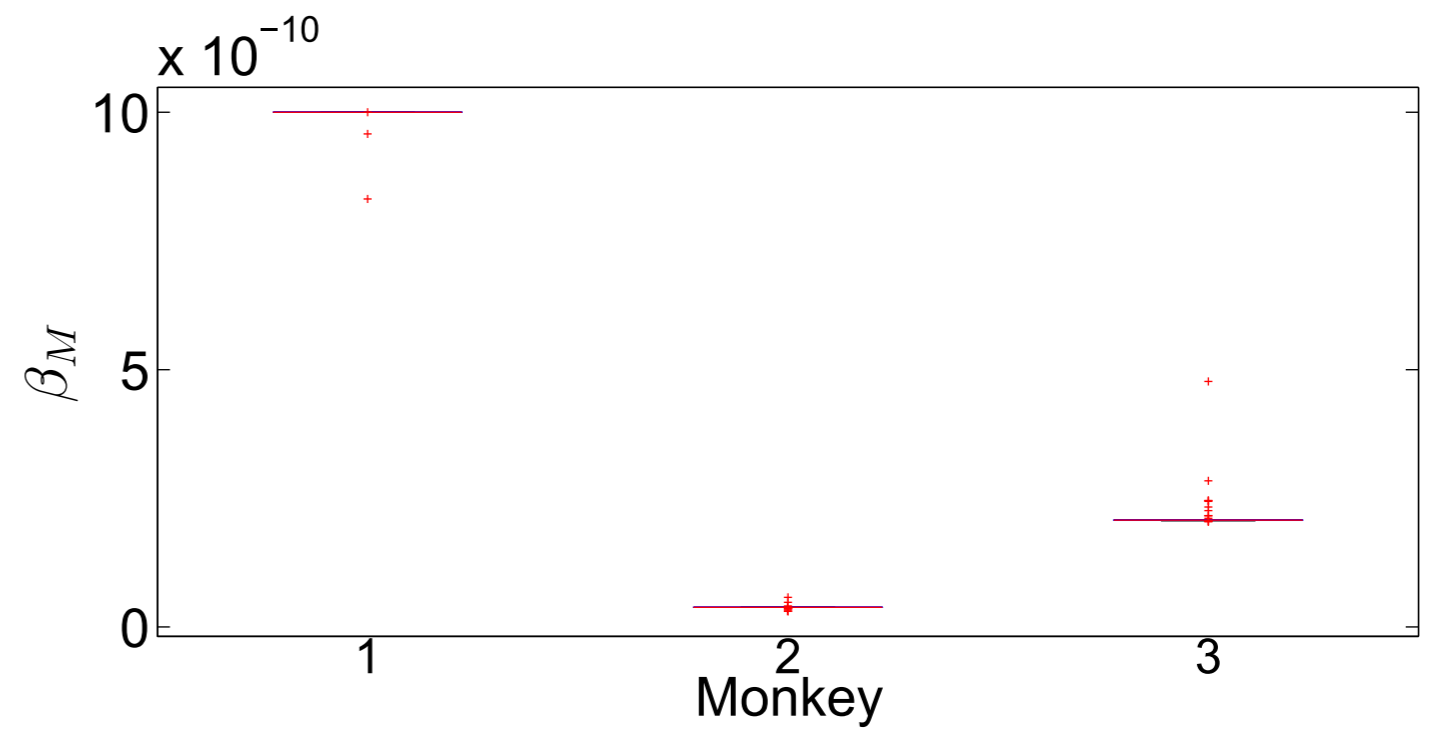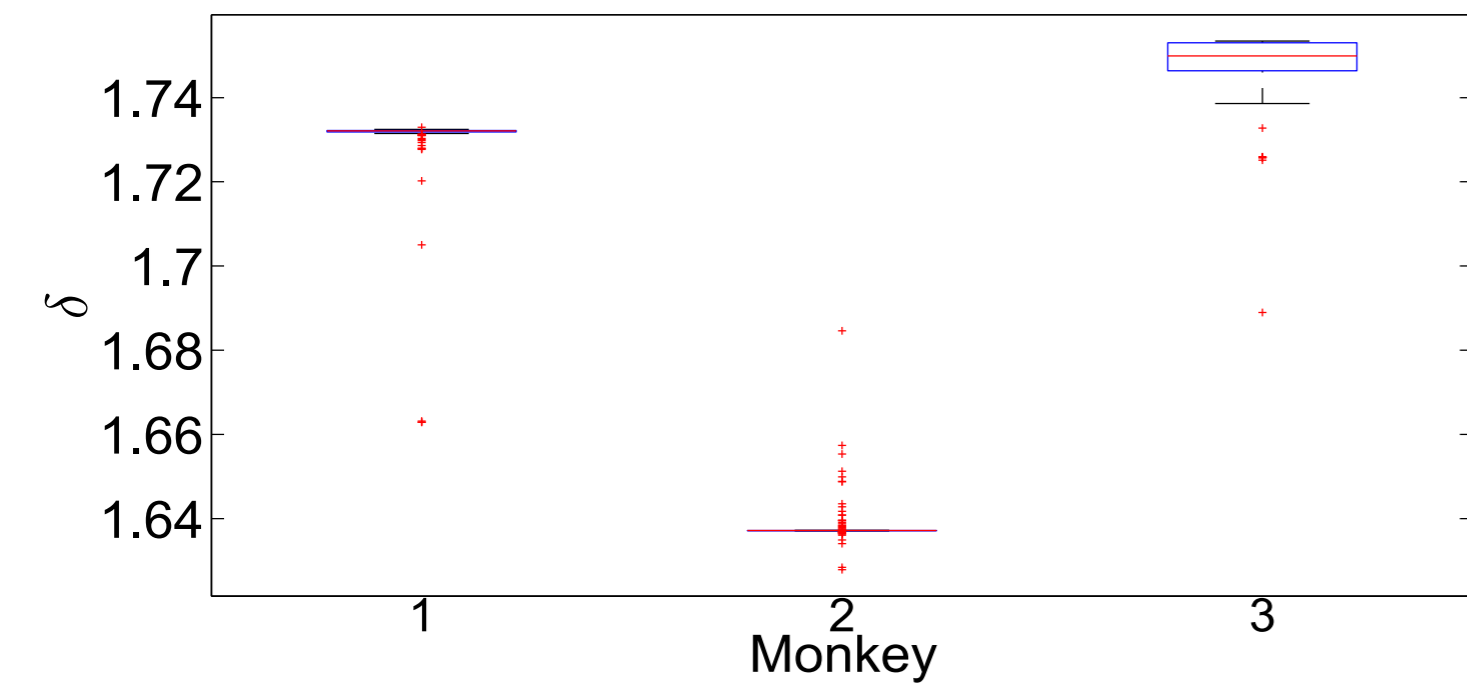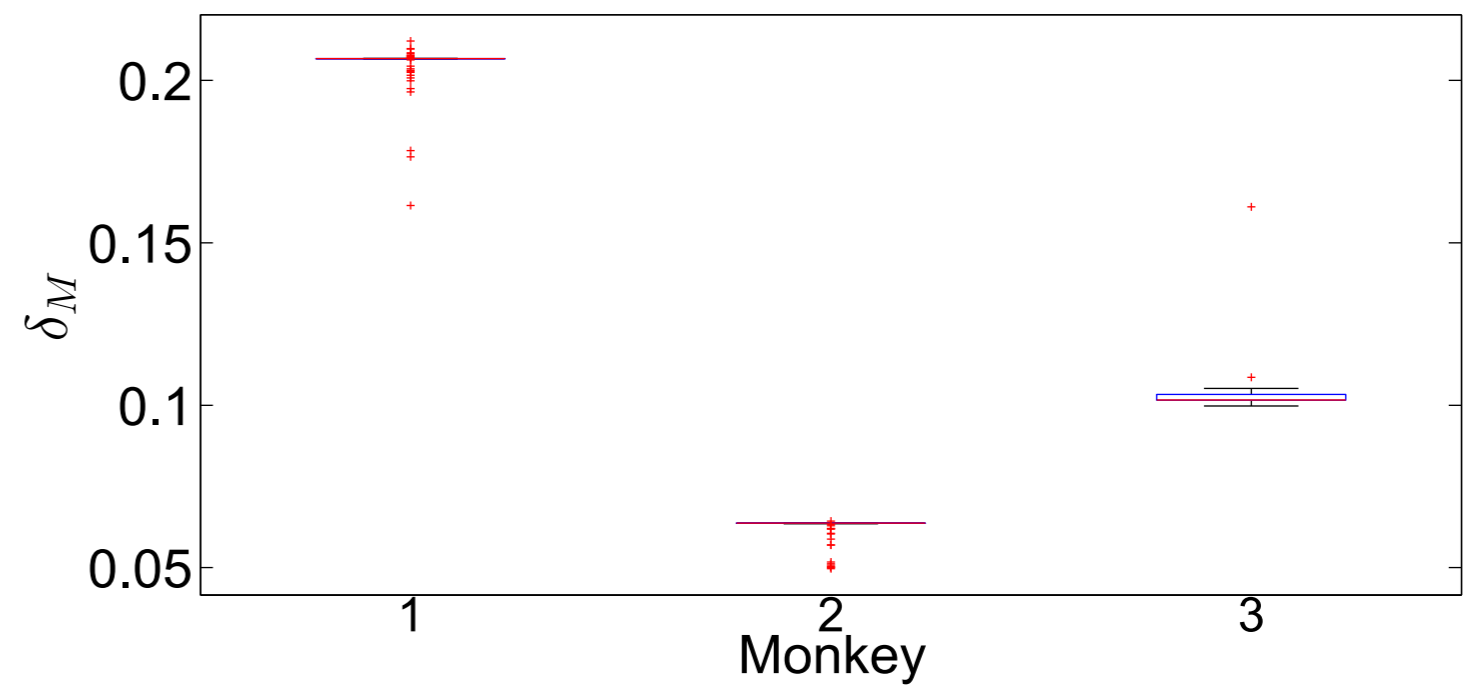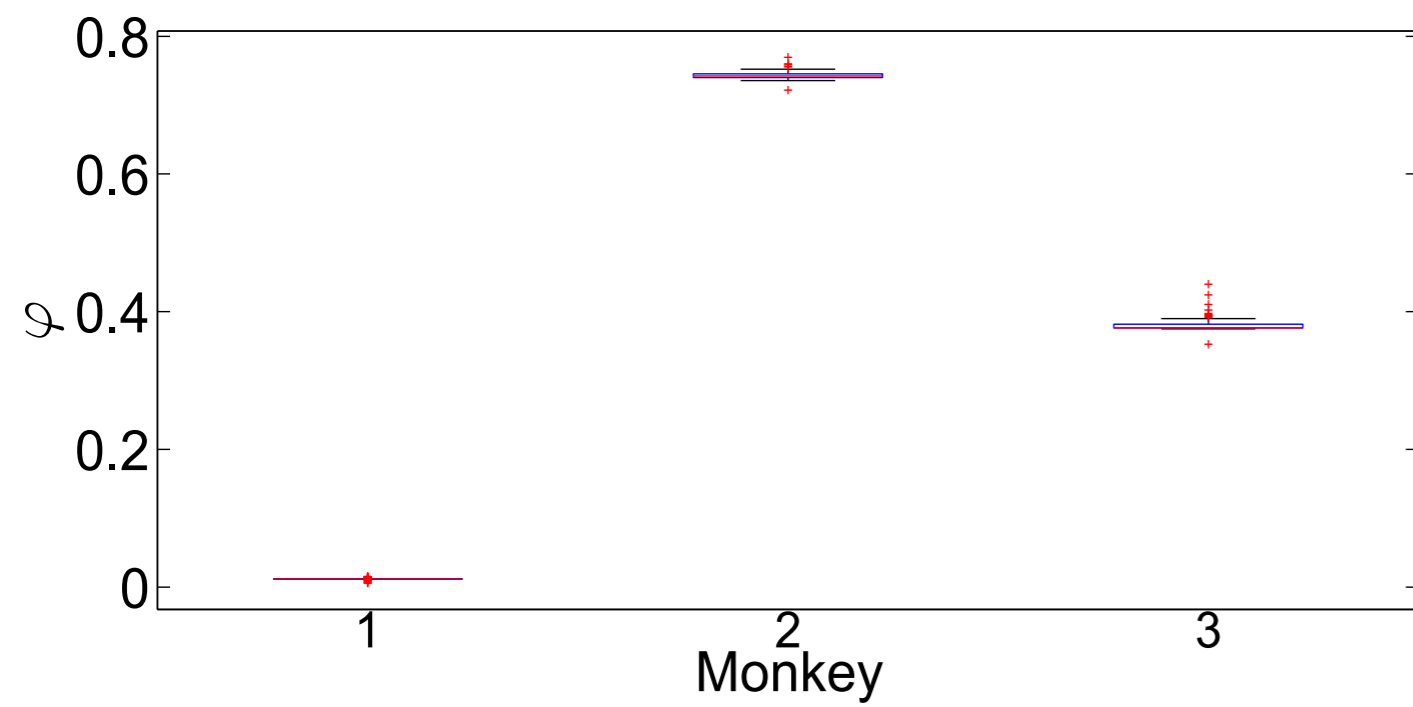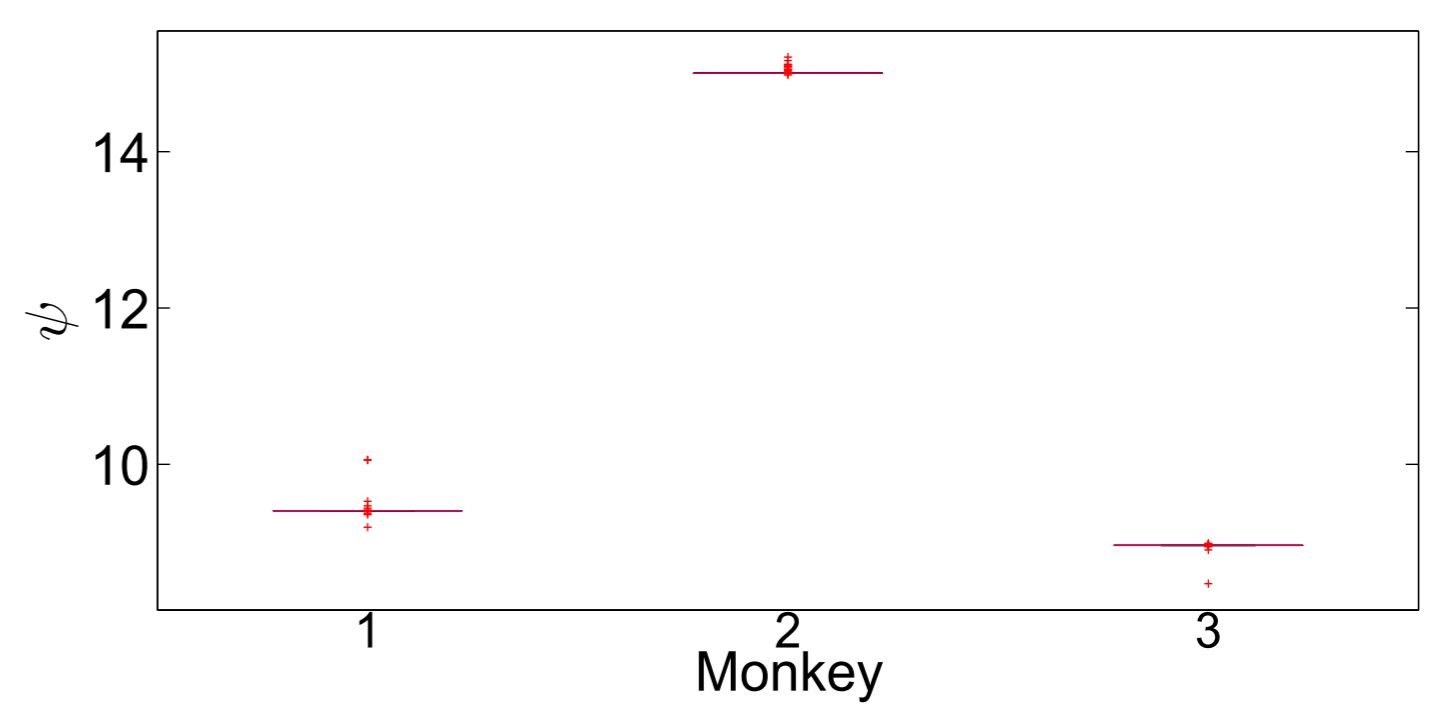

Supplement: S2 Fig — Box-plots of the parameter estimates from 200 data-fittings with values of M0 and MB0 selected randomly from ±10% of the base values. Each subfigure represents the result for one of the parameters estimated. We found that the estimated values remain almost the same when M0, and MB0 were chosen from the range of ±10% of the base values. (PDF) [file pcbi.1008305.s002.pdf]

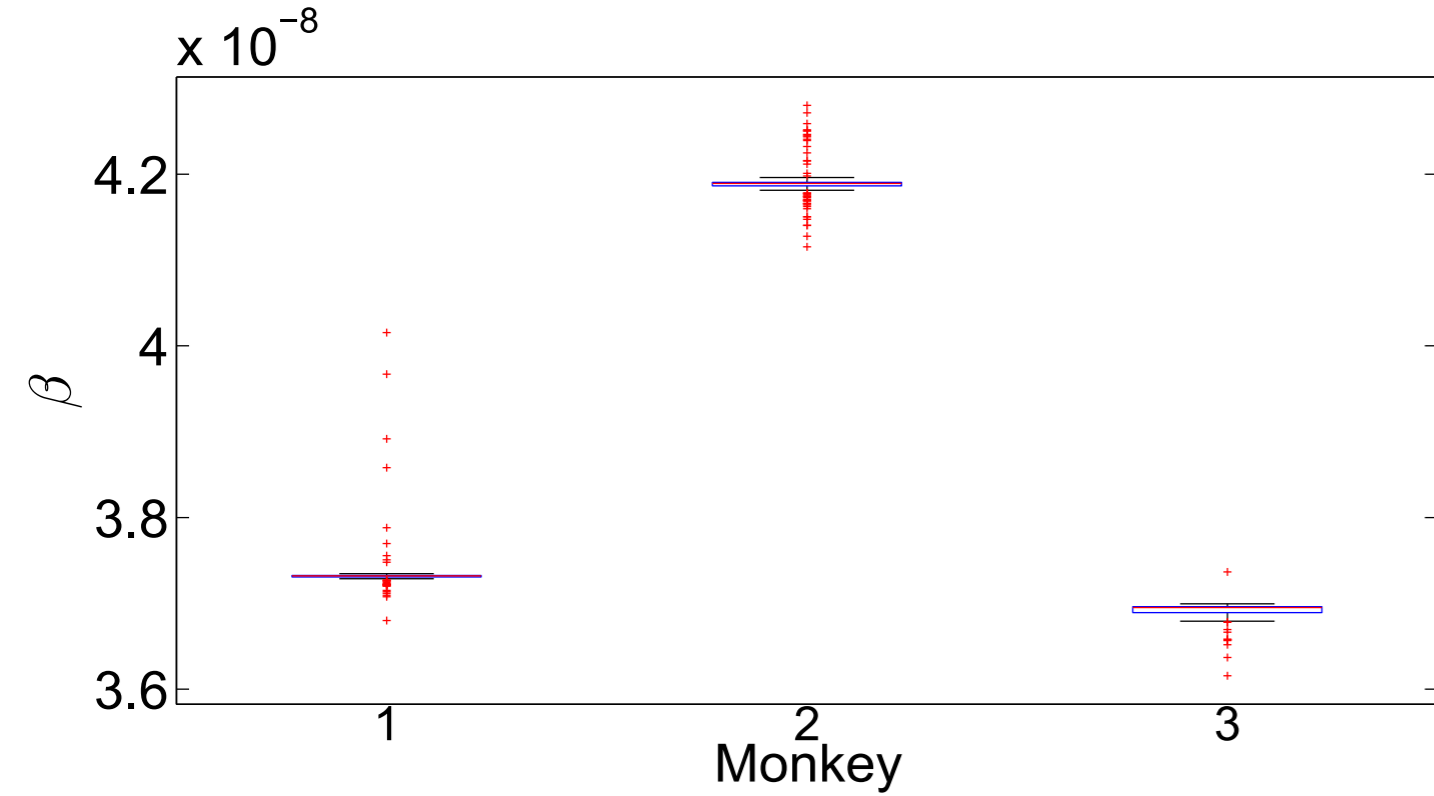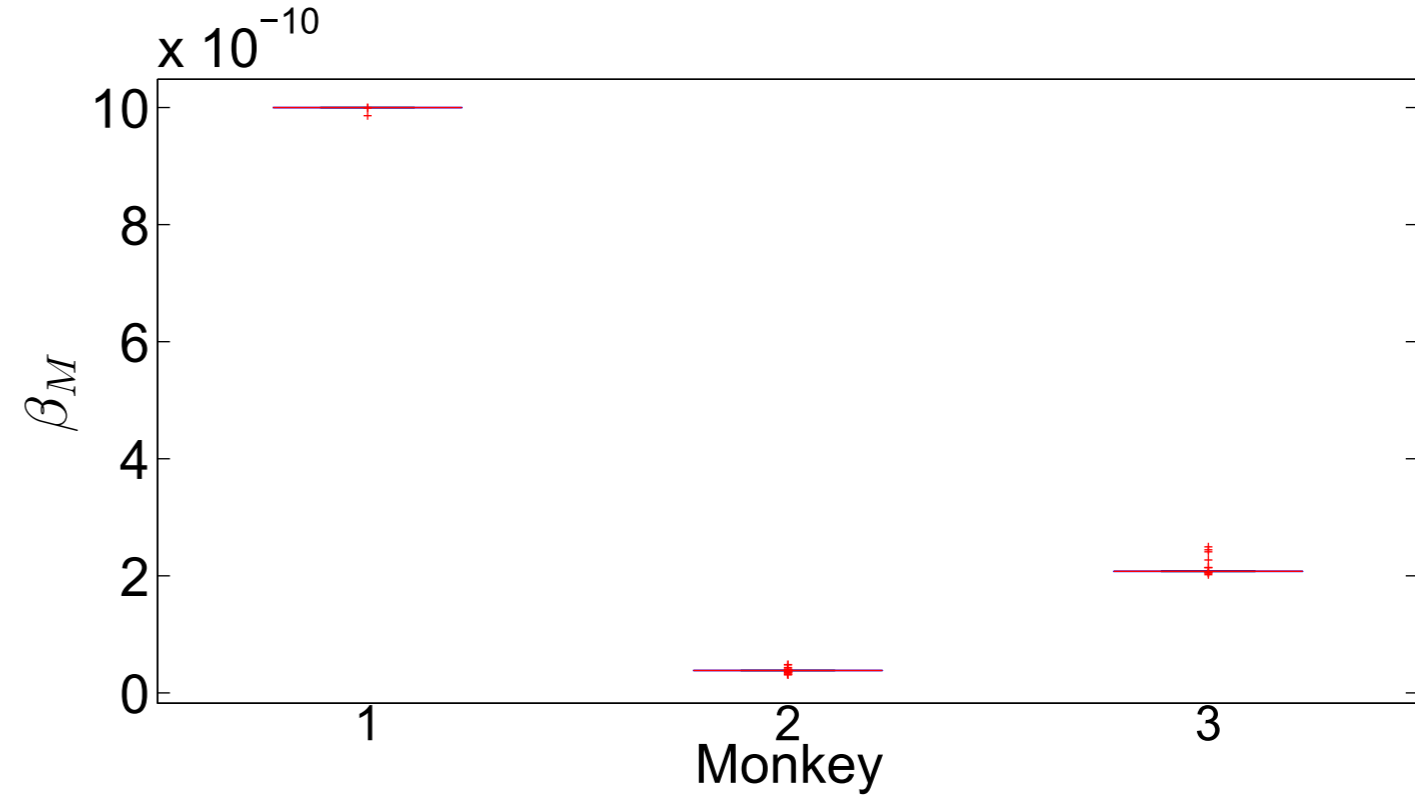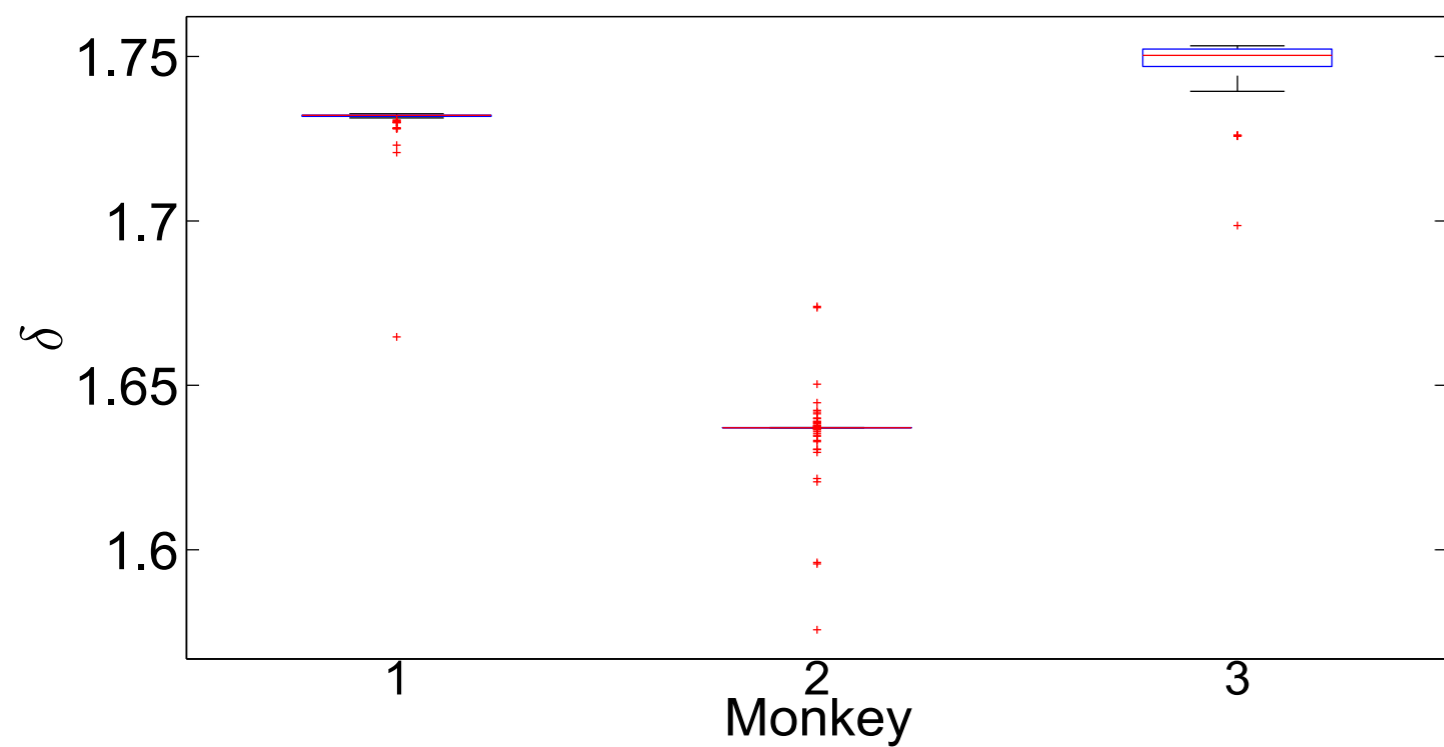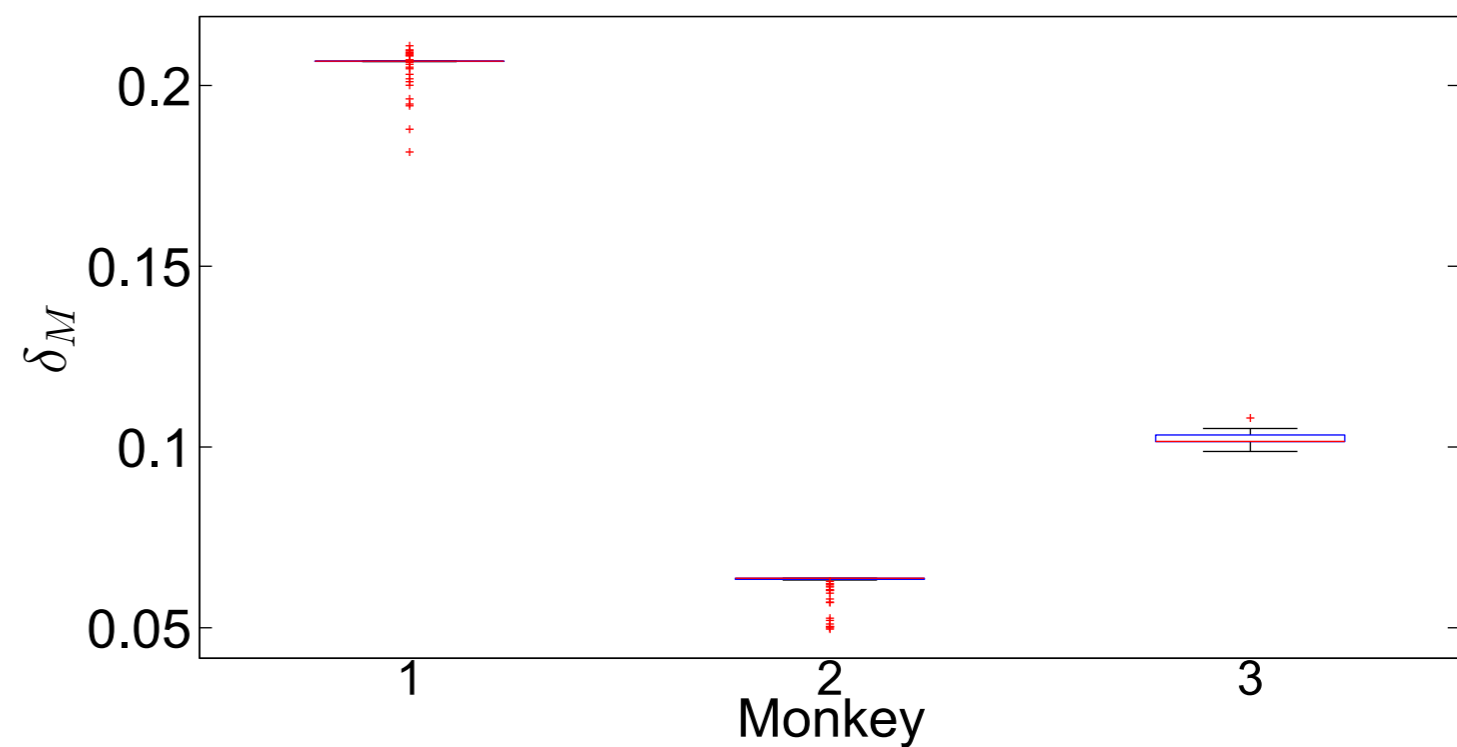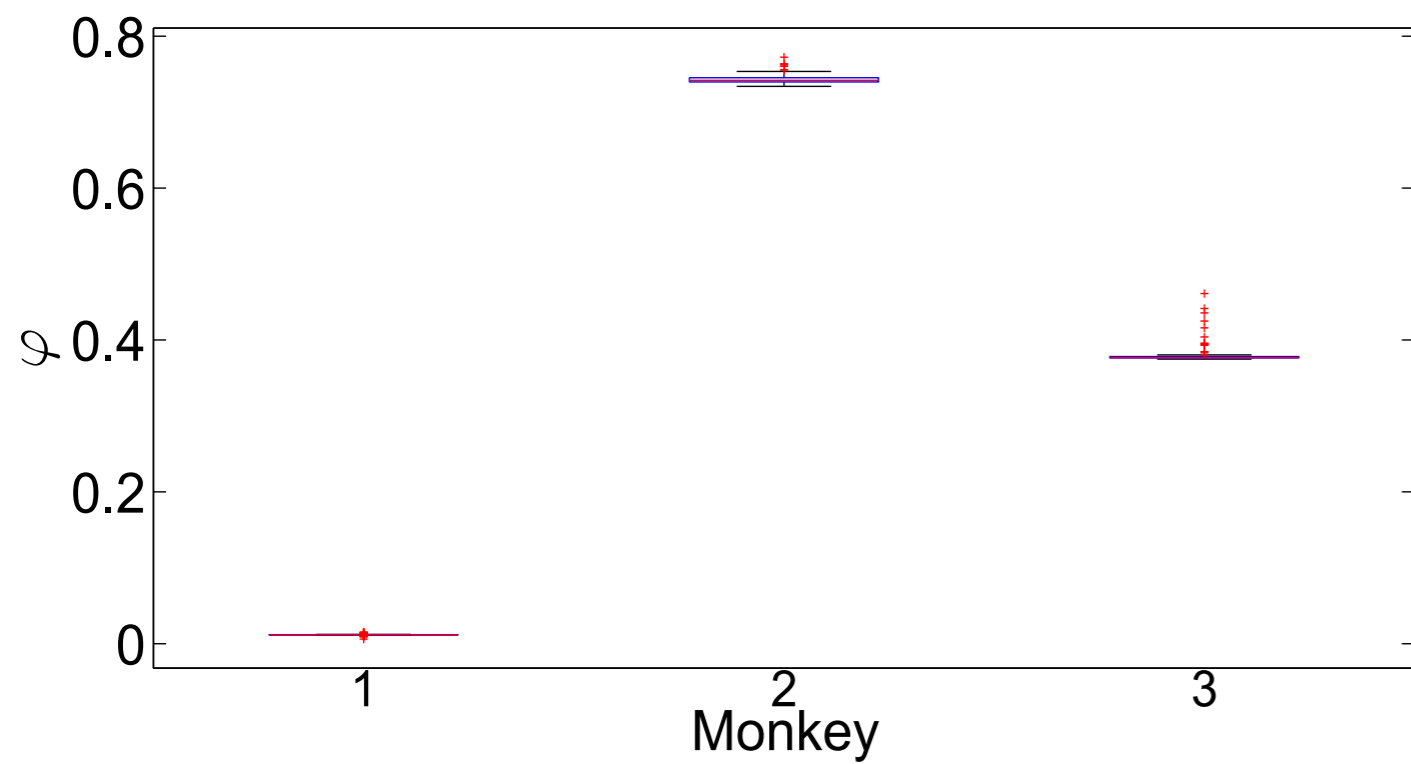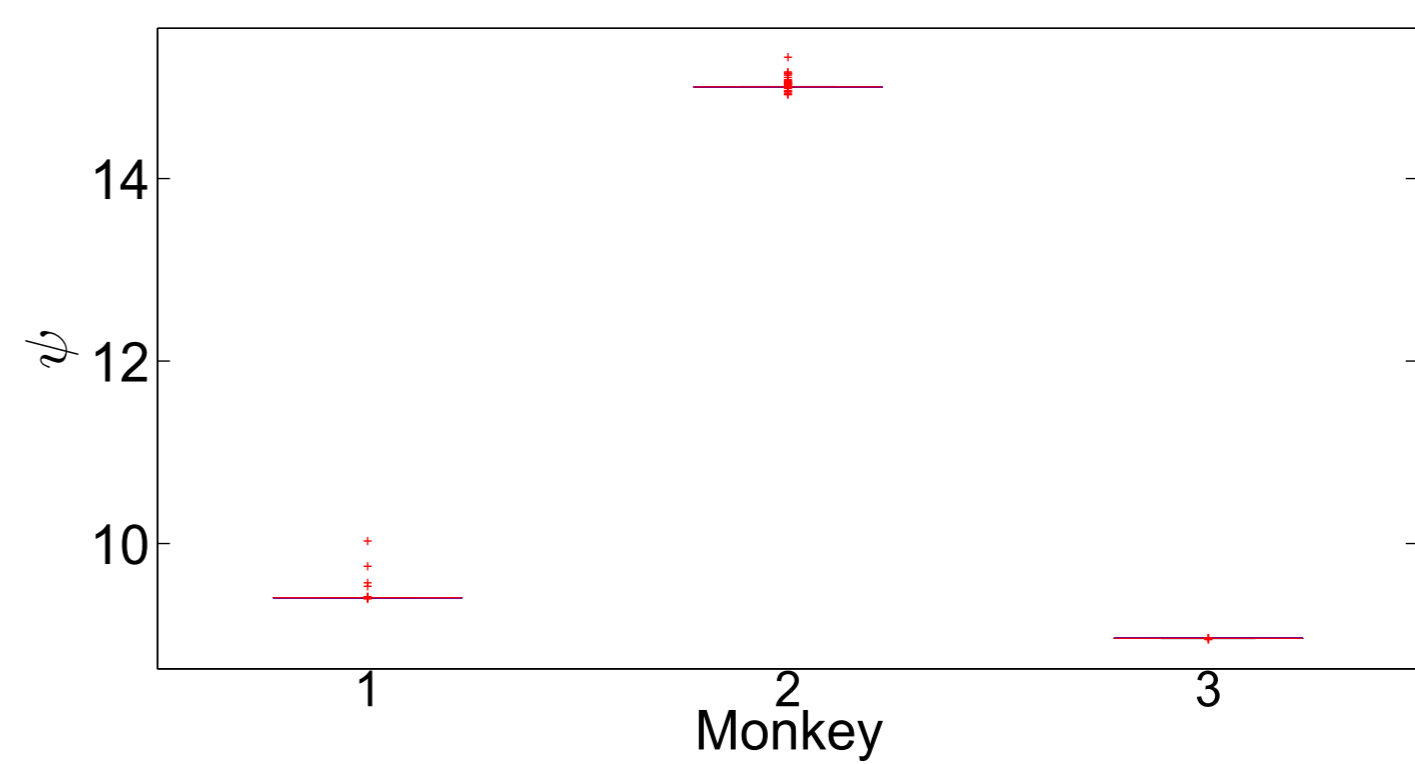

Supplement: S3 Fig — Box-plots of the parameter estimates from 200 data-fittings with values of dM and pM selected randomly from ±10% of the base values. Each subfigure represents the result for one of the parameters estimated. We found that the estimated values remain almost the same when dM and pM were chosen from the range of ±10% of the base values. (PDF) [file pcbi.1008305.s003.pdf]
