# Supplementary material for: Modeling HIV-1 infection in the brain
Source: PLoS Comput Biol. 2020 Nov 19;16(11):e1008305. doi: 10.1371/journal.pcbi.1008305 (PMC7714358; doi:10.1371/journal.pcbi.1008305)
Supplement: S1 Text — (PDF) [file pcbi.1008305.s004.pdf]

Supporting Text - Modeling HIV-1 Infection in the Brain

Colin T. Barker[1,2], Naveen K. Vaidya[3,4,5,*]

1 Department of Mathematics and Computer Science, Drury University, Missouri, USA
2 Department of Mathematics and Statistics, University of Missouri-Kansas City, Missouri, USA
3 Department of Mathematics and Statistics, San Diego State University, San Diego, California, USA
4 Computational Science Research Center, San Diego State University, San Diego, California, USA
5 Viral Information Institute, San Diego State University, San Diego, California, USA

* Corresponding author: nvaidya@sdsu.edu

Derivation of $\mathfrak{R}_0$

Our model possesses a unique infection-free equilibrium (IFE), $(T_*, 0, M_*, 0, M_{B*}, 0, 0, 0)$, where

$$\begin{aligned} T_* &= \frac{\lambda}{d}, \\ M_* &= \frac{\lambda_M(\psi + d_M)}{(\varphi + d_M)(\psi + d_M) - \psi\varphi}, \qquad \text{and} \\ M_{B*} &= \frac{\lambda_M \varphi}{(\varphi + d_M)(\psi + d_M) - \psi\varphi}. \end{aligned}$$

Following the next generation matrix method, we linearize the five model equations corresponding to infection classes, *i.e.*, T^*, M^*, M_B^*, V, and V_B, about the IFE, and introduce the following matrices:

$$F = \begin{bmatrix} 0 & 0 & 0 & \frac{\beta\lambda}{d} & 0 \\ 0 & 0 & 0 & \frac{\beta_M \lambda_M(\psi + d_M)}{(\varphi + d_M)(\psi + d_M) - \psi\varphi} & 0 \\ 0 & 0 & 0 & 0 & \frac{\beta_M \lambda_M \varphi}{(\varphi + d_M)(\psi + d_M) - \psi\varphi} \\ p & p_M & 0 & 0 & 0 \\ 0 & 0 & p_M & 0 & 0 \end{bmatrix},$$

and

$$V = \begin{bmatrix} \delta & 0 & 0 & 0 & 0 \\ 0 & \varphi + \delta_M & -\psi & 0 & 0 \\ 0 & -\varphi & \psi + \delta_M & 0 & 0 \\ 0 & 0 & 0 & c & 0 \\ 0 & 0 & 0 & 0 & c \end{bmatrix},$$

where F represents a matrix of new infections and/or viral production in the linearized system and V represents a matrix of the transfer of cell or virus into and out of the

compartment. The basic reproduction number is then given by the spectral radius of FV^{-1}. That is,

$$\mathfrak{R}_0 = \frac{1}{2\varphi\psi AD} \cdot \left[\sqrt{2}\sqrt{\varphi\psi AD\left(AB + CE + \sqrt{(AB)^2 + HC^2 + C(B+C)G + BC\Psi}\right)}\right],$$

where

$$A = \frac{\varphi^2 + \delta_M\varphi + d_M\varphi + 2\varphi\psi + \psi^2 + \delta_M\psi + d_M\psi + d_M\delta_M}{\varphi\psi}, \qquad B = p\beta\lambda\delta_M d_M\varphi\psi,$$

$$C = p_M\beta_M\lambda_M\delta d\varphi\psi, \qquad D = d_M c\delta d\delta_M, \qquad E = \varphi^2 + 2\delta_M\varphi + \varphi\psi + d_M\psi + d_M\delta_M,$$

$$G = \frac{\varphi^4 + (\varphi\psi)^2 + 2d_M\psi(\varphi^2 + \delta_M\varphi + d_M\delta_M)}{(\varphi\psi)^2}, \qquad H = \frac{1 + \psi^2 - 2d_M\delta_M\varphi^2 + 2d_M\varphi\psi^2}{(\varphi\psi)^2},$$

and

$$\begin{aligned}\Psi =& \frac{1}{(\varphi\psi)^2}\big(-3\varphi^4 - 2\varphi^2\psi + (d_M + \delta_M)(-2\varphi^3 + 2d_M\psi(\psi + \delta_M)) \\ &+ 2(\varphi + d_M)(\psi^3 + d_M\delta_M) + (\varphi\psi)^2 + 2d_M\delta_M(\psi^2 + d_M\delta_M)\big).\end{aligned}$$
