# Supplementary material for: Modeling HIV-1 infection in the brain
Source: PLoS Comput Biol. 2020 Nov 19;16(11):e1008305. doi: 10.1371/journal.pcbi.1008305 (PMC7714358; doi:10.1371/journal.pcbi.1008305)
Supplement: S2 Text — (PDF) [file pcbi.1008305.s005.pdf]

Supporting Text - Modeling HIV-1 Infection in the Brain

Colin T. Barker[1,2], Naveen K. Vaidya[3,4,5,*]

1 Department of Mathematics and Computer Science, Drury University, Missouri, USA
2 Department of Mathematics and Statistics, University of Missouri-Kansas City, Missouri, USA
3 Department of Mathematics and Statistics, San Diego State University, San Diego, California, USA
4 Computational Science Research Center, San Diego State University, San Diego, California, USA
5 Viral Information Institute, San Diego State University, San Diego, California, USA

* Corresponding author: nvaidya@sdsu.edu

Results from Model 2

We used Model 2 to compute the dynamics of incoming infected macrophages into the brain (Fig A, left). The time-dependent nature of this dynamics is preserved in Model 2 predictions as well, but the magnitude is lower than that predicted by Model 1. For example, the peak infected macrophages entering the brain predicted by Model 2 is 3 times lower than Model 1. Moreover, we used Model 2 to observe the long-term simulations (1000 days post-infection) of the viral load in the brain and in the plasma (Fig A, right) and found similar dynamics as predicted by Model 1. From a detailed analysis of Model 2, we found that although there are some slightly different quantitative results (for instance, the median values of BBB transport rate were estimated to be $\phi = 0.2916$ and $\psi = 13.19$ for Model 2 vs. $\phi = 0.2915$ and $\psi = 9.962$ for Model 1), the overall behavior of Model 2 is similar to that of Model 1.

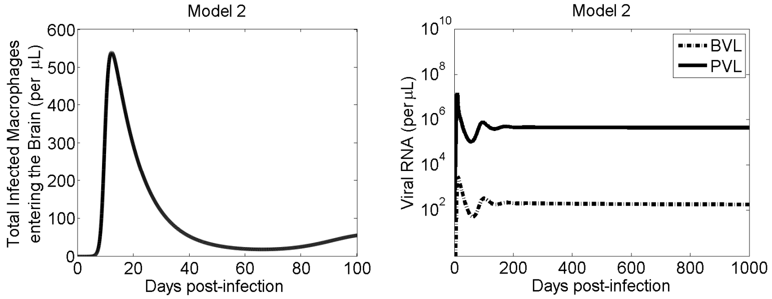

Fig A. Results from Model 2. Model 2 prediction of the total count of infected macrophages (φM^*) entering the brain for 100 days post-infection (left) and the long-term plasma and brain viral dynamics for 1000 days post-infection (right).
