# Supplementary material for: Modeling HIV-1 infection in the brain
Source: PLoS Comput Biol. 2020 Nov 19;16(11):e1008305. doi: 10.1371/journal.pcbi.1008305 (PMC7714358; doi:10.1371/journal.pcbi.1008305)
Supplement: S3 Text — (PDF) [file pcbi.1008305.s006.pdf]

Supporting Text - Modeling HIV-1 Infection in the Brain

Colin T. Barker[1,2], Naveen K. Vaidya[3,4,5,*]

1 Department of Mathematics and Computer Science, Drury University, Missouri, USA
2 Department of Mathematics and Statistics, University of Missouri-Kansas City, Missouri, USA
3 Department of Mathematics and Statistics, San Diego State University, San Diego, California, USA
4 Computational Science Research Center, San Diego State University, San Diego, California, USA
5 Viral Information Institute, San Diego State University, San Diego, California, USA

* Corresponding author: nvaidya@sdsu.edu

Viral Dynamics under HAART

To study the impact of virus replication and viral production inside the brain under HAART, we performed model simulations by introducing HAART after both brain and plasma viral load reach steady state ($\sim$ 250 days post-infection). To mimic the situation in which the antiretroviral drugs fail to cross the BBB, we completely suppressed the viral production outside the brain (i.e. $p = p_M = 0$ outside the brain) and preserved ongoing viral replication and viral production inside the brain (i.e., $p_M > 0$ inside the brain). Our model predicts that for low level of viral productions in the brain, both plasma and brain viral loads remain undetected. However, for sufficiently large viral productions inside the brain, the brain sustains a significantly high viral load, while the plasma viral load remains undetected (Fig A). Therefore, it is possible that the virus replication and virus production inside the brain constitute an important factor for virus persistence despite the successful suppression of virus outside the brain.

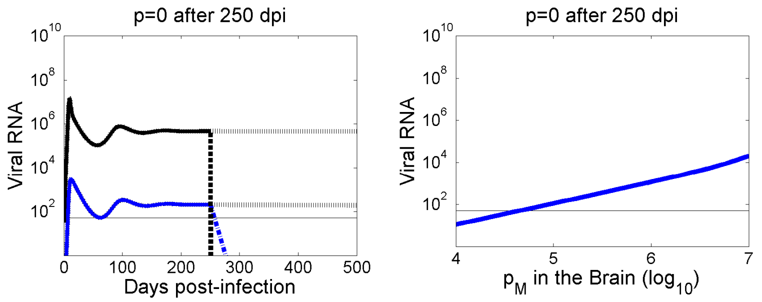

Fig A. Model simulation under HAART. (Left) Plasma viral load (black) and CSF viral load (blue) predicted by Model 1 under HAART initiated at the steady state. (Right) Brain viral load and plasma viral load months after HAART is initiated. Note that the plasma viral load is completely suppressed, so it does not appear in the right graph.
